# Improved Plant Nitrate Status Involves in Flowering Induction by Extended Photoperiod

**DOI:** 10.3389/fpls.2021.629857

**Published:** 2021-02-12

**Authors:** Jia Yuan Ye, Wen Hao Tian, Miao Zhou, Qing Yang Zhu, Wen Xin Du, Chong Wei Jin

**Affiliations:** ^1^State Key Laboratory of Plant Physiology and Biochemistry, College of Natural Resources and Environmental Science, Zhejiang University, Hangzhou, China; ^2^State Key Laboratory of Plant Physiology and Biochemistry, College of Life Science, Zhejiang University, Hangzhou, China

**Keywords:** nitrogen, photoperiodic flowering responses, suboptimal nitrate supply, *NRT1.1*, *NRT2.1*, *FLOWERING LOCUS T*, *CONSTANS*, nitrate uptake

## Abstract

The floral transition stage is pivotal for sustaining plant populations and is affected by several environmental factors, including photoperiod. However, the mechanisms underlying photoperiodic flowering responses are not fully understood. Herein, we have shown that exposure to an extended photoperiod effectively induced early flowering in *Arabidopsis* plants, at a range of different nitrate concentrations. However, these photoperiodic flowering responses were attenuated when the nitrate levels were suboptimal for flowering. An extended photoperiod also improved the root nitrate uptake of by NITRATE TRANSPORTER 1.1 (NRT1.1) and NITRATE TRANSPORTER 2.1 (NRT2.1), whereas the loss of function of NRT1.1/NRT2.1 in the *nrt1.1-1/2.1-2* mutants suppressed the expression of the key flowering genes *CONSTANS* (*CO*) and *FLOWERING LOCUS T* (*FT*), and reduced the sensitivity of the photoperiodic flowering responses to elevated levels of nitrate. These results suggest that the upregulation of root nitrate uptake during extended photoperiods, contributed to the observed early flowering. The results also showed that the sensitivity of photoperiodic flowering responses to elevated levels of nitrate, were also reduced by either the replacement of nitrate with its assimilation intermediate product, ammonium, or by the dysfunction of the nitrate assimilation pathway. This indicates that nitrate serves as both a nutrient source for plant growth and as a signaling molecule for floral induction during extended photoperiods.

## Introduction

The transition from vegetative to reproductive development is a pivotal event in the lives of annual plants and can have a profound impact on their fertility and population sustainability (Guo and Yang, 1998; Lin and Tsay, 2017). Floral induction is regulated by both environmental and endogenous cues. The isolation of loss-of-function mutations and the analysis of transgenic plants, has resulted in hundreds of genes that are related to the timing of flowering being classified into several distinct pathways in *Arabidopsis thaliana*, including photoperiod, vernalization, gibberellin acid, autonomous, age, sugar, and temperature pathways (Fornara et al., 2010; Srikanth and Schmid, 2011; Kim et al., 2012; Wahl et al., 2013; Capovilla et al., 2014; Yuan et al., 2016; Luo et al., 2018; Andrés et al., 2020; Bao et al., 2020). These pathways were found to converge on the “integrator” genes *SUPPRESSOR OF OVEREXPRESSION OF CO 1* (*SOC1*) and *FLOWERING LOCUS T* (*FT*) (Putterill et al., 1995; Luo et al., 2018; Andrés et al., 2020). Among the environmental cues, photoperiod, which integrates inputs from the circadian clock and light receptors, is the most important seasonal cue in floral induction (Bao et al., 2019). In most cases, angiosperms flower earlier in extended photoperiod conditions (Andrés and Coupland, 2012; Luo et al., 2018; Bao et al., 2019). Several studies have indicated that the flowering of *Arabidopsis* induced by the long-day (LD) treatments is under the control of the canonical genetic photoperiod pathway (Samach et al., 2000; Suárez-López et al., 2001; Fornara et al., 2010; Andrés and Coupland, 2012). Briefly, the FLAVIN-BINDING KELCH REPEAT F-BOX 1 (FKF1) perceives light signals and interacts with GIGANTEA (GI) to mediate the degradation of CONSTANS (CO) transcriptional repressors CYCLING DOF FACTORs (CDFs), which promote *CO* transcripts (Sawa et al., 2007; Fornara et al., 2009, 2010). The CO protein then interacts with the B and C subunits of Nuclear Factor Y (NF-Y) to form the NF-CO complex, which increases *FT* expression and thus initiates flowering (Wenkel et al., 2006; Tiwari et al., 2010; Andrés and Coupland, 2012; Gnesutta et al., 2017; Luo et al., 2018; Bao et al., 2019). Floral repressors are also involved in the regulation of photoperiodic flowering responses. For example, the AP2-like transcription factor SCHLAFMUTZE (SMZ) and its paralog, SCHNARCHZAPFEN (SNZ) delay flowering under LD conditions. Furthermore, chromatin immunoprecipitation experiments have shown that SMZ can directly bind to the FT locus, which leads to the downregulation of *FT* expression (Mathieu et al., 2009; Golembeski and Imaizumi, 2015).

In addition to the photoperiod, the availability of nitrogen (N), which is an integral mineral element for plants and required in quantities higher than any other, is an important environmental factor that affects flowering time (Martínez et al., 2004; Achard et al., 2006; Castro Marín et al., 2011; Kant et al., 2011; Liu et al., 2013; Yuan et al., 2016; Zeng et al., 2017; Gras et al., 2018; Olas et al., 2019). The effects of nitrate on flowering have been intensively studied in recent decades. By summarizing previous studies and further examining the flowering time of *Arabidopsis* plants with various additional levels of nitrate, Lin and Tsay (2017) propose a U-shaped response of flowering to nitrate. They identified that an optimal level of nitrate facilitates flowering and that nitrate levels above or below this delayed flowering. Although further studies may be required to clarify whether the U-shaped response of flowering to nitrate can be generalized for natural conditions, it could be reasonably suspected that the factors affecting root nitrate acquisition may also influence flowering. Several studies have indicated that carbohydrate photosynthates, such as sucrose and trehalose-6-phosphate (T6P), promote flowering (Ohto et al., 2001; Gómez et al., 2010; Moghaddam and van den Ende, 2013; Wahl et al., 2013; Andrés et al., 2020). Very recently, Olas et al. (2019) found that the knockdown of *TREHALOSE PHOSPHATE SYNTHASE 1*, which encodes the enzyme producing T6P, was unable to flower under limited N soil conditions but could flower under optimal N soil and short-day (SD) conditions, highlighting the combined importance of T6P and nitrate for floral induction. Interestingly, light conditions intensively affect nitrate acquisition by the roots. For example, nitrate uptake by soybean and barley plants could be increased by 150% and 300%, respectively, under light conditions compared to dark conditions (Delhon et al., 1995; Peuke and Jeschke, 1998; Lejay et al., 1999). Furthermore, it was shown that the loss of function of a light-responsive bZIP transcription factor ELONGATED HYPOCPTYL5 (HY5) in *Arabidopsis hy5* mutants, suppressed nitrate uptake by decreasing the level of carbon photo-assimilate sucrose (Chen et al., 2016). In contrast, the exogenous application of sucrose prevented the inhibition of *NRT1.1* and *NRT2.1* expression under dark conditions, thus significantly increasing nitrate uptake in the dark (Lejay et al., 1999, 2003). Theoretically, changes in the photoperiod could be expected to alter the production of carbon photo-assimilates in plants and may subsequently modify the nitrate uptake by roots. In this context, the mechanisms underlying the photoperiodic flowering responses are probably associated with alterations in the uptake of nitrate.

In well-aerated soils, nitrate is the primary N source, owing to rapid nitrification (Miller and Cramer, 2005), and the concentration of nitrate varies greatly in different soil environments, ranging from 0 to 1.8 mM, and even above 70 mM (Crawford and Glass, 1998). However, in most natural habitats, such as forest and grassland ecosystems, soil nitrate concentrations are <1 mM. In agricultural ecosystems, nitrate concentrations in soils are typically 1–5 mM, but insufficient N levels are common due to the rapid depletion of nitrate from soil solutions (Crawford and Glass, 1998; Miller and Cramer, 2005). Therefore, it is important to clarify if nitrate uptake plays a role in regulating photoperiod-modulated flowering when nitrate supplies are below the optimal levels, to more reasonably evaluate the impact of nitrate on the plant ecosystem and crop production. In this study, we investigated the above notion and demonstrated that the nitrate status of the soil is involved in photoperiod-modulated flowering when it is below the optimal level. Specifically, extended photoperiod conditions improved root nitrate uptake by NRT1.1 and NRT2.1, which subsequently upregulated the expression of the key light-responsive regulators, *FT* and *CO*, in plants, thereby contributing to premature flowering induction.

## Experimental Procedures

### Plant Material

The *Arabidopsis* mutants *nrt1.1-1* (Salk _097431), *nrt2.1-2* (CS859604), and *nia2-5* (CS2355) were obtained from the *Arabidopsis* Biological Resource Center. The *ft-10* and *co-1* mutants were kindly provided by Y.R. Hu (Xishuangbanna Tropical Botanical Garden, Chinese Academy of Sciences, China), and the *cry1* mutant (CS6955) were kindly provided by S. Yuan (Sichuan Agricultural University, China) and B. Liu (Chinese Academy of Agricultural Sciences, Beijing, China). All mutants were all in Columbian (Col-0) background. The *nrt1.1-1/2.1-2* mutants were generated by crossing *nrt1.1-1* with *nrt2.1-2*, as described previously (Ye et al., 2019). As nitrate storage in seeds may affect their germination time, we prolonged the vernalization period to minimize the differences in the germination times between the different plant lines. Consequently, all the seeds used in this study were harvested at the same time and stored for approximately 1 year at 4°C; furthermore, another 3 days of stratification were also conducted at 4°C before sowing the seeds.

### Plant Culture and Treatment Conditions

For agar culturing, the surface-sterilized *Arabidopsis* seeds were sown on basal agar medium containing 1% (w/v) sucrose, 0.8% agar (w/v) (Sigma), NaH_2_PO_4_ (1,000 μM), MgSO_4_ (500 μM), CaCl_2_ (1,000 μM), H_3_BO_3_ (10 μM), MnSO_4_ (0.5 μM), ZnSO_4_ (0.5 μM), CuSO_4_ (0.1 μM), (NH_4_)_6_Mo_7_O_24_ (0.1 μM), and Fe-EDTA (25 μM). Different concentrations of KNO_3_ ranging from 0.2 mM to 16 mM were supplied to the agar medium as the nitrogen source according to the different experimental requirements, and the resulting differences in K concentrations were balanced by adjusting the K_2_SO_4_ concentration, as indicated in the figure legends. To clarify whether nitrate functions as a nutrient source to control photoperiod-modulated flowering, different concentrations of (NH_4_)_2_SO_4_ including 0.2, 0.5, 1.0, and 2.0 mM were used to replace KNO_3_ in the agar medium, and the K concentrations in all (NH_4_)_2_SO_4_ treatments were adjusted to 2 mM using K_2_SO_4_. The pH was adjusted to 6.0. The plants were transferred to a growth room where they were maintained under photoperiod cycles of 16 h light/8 h dark (LD) or 8 h light/16 h dark (SD). The light intensity and temperature were maintained at 100 μmol m^–2^ s^–1^ and 22°C, respectively. Bolting time (days after germination [DAG]) and rosette leave numbers were determined when the main inflorescence had reached a height of 0.5 cm, while flowering time (DAG) was determined when the first flower was visible.

For soil culturing, the plants were grown in square pots (6 cm in length and 6 cm in height) containing soil:perlite at a ratio of 1:1.5, under LD (16-h light/8-h dark) conditions. The soil and perlite were autoclaved prior to use to kill insect eggs. Seed stratification was conducted in 0.05% agar at 4°C for 3 days, before planting. In each pot, 4–8 wild-type seeds were sown, and a single seedling was retained at around 7 DAG. The plants were watered twice a week with approximately 20 mL of medium per pot, per watering event. Different concentrations of KNO_3_ including 1, 2, 3, 6, 10, and 20 mM were supplied to the medium as the nitrogen source, and the resulting differences in the K concentrations were balanced by adjusting the K_2_SO_4_ concentration. The composition of other nutrients in the medium for the soil system was as follows: NaH_2_PO_4_ (1,000 μM), MgSO_4_ (500 μM), CaCl_2_ (1,000 μM), H_3_BO_3_ (10 μM), MnSO_4_ (0.5 μM), ZnSO_4_ (0.5 μM), CuSO_4_ (0.1 μM), (NH_4_)_6_Mo_7_O_24_ (0.1 μM), and Fe-EDTA (25 μM).

### Measurement of the NO_3_*^–^* Uptake

The *Arabidopsis* plants were grown hydroponically for 2 weeks under either LD or SD conditions with 1 mM nitrate as the exclusive N source. The nutrient solutions were renewed every second day. The daily nitrate uptake rate by the roots was then evaluated using ^15^NO_3_^–^ under the same conditions used for plant growth. Uniform plants grown on a nutrient medium containing 1 mM KNO_3_ had their medium replaced with K^15^NO_3_ (atom% ^15^N, 99%) for 24 h, and were then harvested. The atom% ^15^N was measured in an isotope ratio mass spectrometer (Isorime100; Elementar Analysensysteme, Hanau, Germany). Experiments were replicated four times for each treatment.

A non-invasive microelectrode ion flux measurement system (ipa-2; AE, United States) was used to measure the net fluxes of NO_3_^–^, as previously described (Hawkins et al., 2008; Zhu et al., 2019). Col-0 plants were grown in agar medium under either LD or SD conditions. Nitrate (1 mM) was used as the exclusive N source and seedlings (at the stage when four leaves were visible) were used to determine the transmembrane NO_3_^–^ fluxes of the roots in the mature zone, in the same medium used for plant growth, using a NO_3_^–^-selective microelectrode.

### Quantitative Real-Time Polymerase Chain Reaction

The expression of NO_3_^–^ uptake- and assimilation-related genes was determined using root samples. The expression of flowering-related genes and stress-related marker genes was determined using the shoot samples. Approximately 100 mg of tissue sample was ground in liquid nitrogen, and total RNA was extracted using RNAiso-Plus (TaKaRa, Otsu, Japan). The first-strand cDNA was synthesized using ReverTra Ace qPCR RT Master Mix with gDNA Remover (TOYOBO, Osaka, Japan). SYBR^®^ Green Real-time PCR Master Mix (TOYOBO, Osaka, Japan) was then used to detect the transcript levels of the corresponding genes. The gene-specific primers used in the quantitative Real-Time PCR are listed in Supplementary Table 1. Relative transcript levels were measured, and corrected efficiency calculations were conducted, as described by Fang et al. (2016).

### Grafting of *Arabidopsis* Plants

Grafting between the *nrt1.1-1/2.1-2* mutants and Col-0 plants was performed using a previously described method (Marsch-Martínez et al., 2013). Briefly, plants were sown on basal media containing 1 mM nitrate, and 5-day-old seedlings were used for grafting. After the cotyledons were removed, the hypocotyls were cut horizontally, and the scions were immediately placed into the rootstock. The successfully grafted plants were vertically placed on basal media containing 1 mM nitrate under LD or SD conditions for flowering time measurements.

### Statistical Analysis

Statistical analysis was performed using the SPSS statistical 20.0 software. One-way analysis of variance (ANOVA) with Duncan’s multiple range test was used and significant differences among the treatments were indicated with *P* < 0.05. Interaction differences were tested using a two-way ANOVA. The regression lines were compared using univariate analysis of variance (UNIANOVA), in which the concentrations of the nitrate were the covariates and the genotype (Col-0 or *nrt1.1-1/2.1-2*) was the fixed factor, and △ days to flowering was the dependent variable.

## Results

### Flowering Was Promoted in LD and SD Photoperiods When the Suboptimal Nitrate Supplies Were Elevated

**FIGURE 1:**
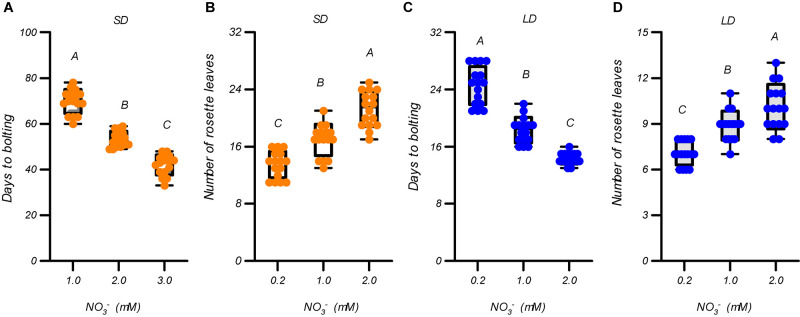
Flowering promotion due to increases in the suboptimal nitrate supply under both LD and SD photoperiods. Days to bolting **(A)** and number of rosette leaves **(B)** of Col-0 plants grown under LD conditions (16-h light/8-h dark). Days to bolting **(C)** and number of rosette leaves **(D)** of Col-0 plants grown under SD conditions (8-h light/16-h dark). Seedlings were planted in media containing 0.2, 1, or 2 mM nitrate. The potassium concentrations in all growth media were adjusted to 2 mM using K_2_SO_4_. At least 20 plants grown in each of the LD and SD conditions were used to determine the bolting time and number of rosette leaves. Error bars represent ± standard deviations. Different letters indicate significant differences between means, as determined using a one-way ANOVA followed by Tukey’s multiple comparisons test (*P* < 0.05).

We first evaluated the effects of different nitrate supplies (∼1 mM) on flowering in *Arabidopsis thaliana* ecotype Col-0 plants to clarify the effects of nitrate availability on flowering under the growth conditions used in this study. Three nitrate levels, 0.2, 1.0, and 2.0 mM, were used. As shown in Figure 1 and Supplementary Figure 1, the elevated nitrate concentrations led to earlier flowering and more rosette leaves under both LD and SD photoperiod conditions. Furthermore, the growth of the plants was improved by increasing the nitrate concentrations under both LD and SD photoperiods (Supplementary Figure 2). We then evaluated the effects of nitrate concentrations >2 mM on the flowering of the plants. In contrast to the above results, the days to flowering under SD conditions were delayed, but the number of rosette leaves increased with an increase in nitrate supply (Supplementary Figure 3). Under LD conditions, the flowering of the plants was also delayed when the nitrate was increased from 2 mM to 4 mM, but further increases above 4 mM had little effect on the delay of flowering and the number of rosette leaves. These results suggest that the flowering of *Arabidopsis* plants is promoted by increasing the nitrate availability, but only below an optimal level, under both LD and SD conditions. Supplementary Figure 3 clearly indicates that the optimal nitrate concentration for flowering was ∼2 mM in our agar-growth system. Interestingly, a linear fit between the flowering time and nitrate concentrations of <2 mM, generated an excellent correlation coefficient for both LD and SD conditions (*R* = 0.95 for LD and 0.96 for SD). This illustrates the linear dependence of flowering time on nitrate availability, when its level is suboptimal for the plants, under both LD and SD conditions. We also measured the effects of different nitrate concentrations (1 to 20 mM) on the flowering of plants grown in a soil-based system under LD photoperiod condition. As shown in Supplementary Figure 4, the flowering time along with the elevation in nitrate supply in a soil-based system, showed a similar pattern to that in the agar-based growth system (Supplementary Figure 3). This indicated that the acceleration in flowering observed with the elevation in nitrate availability, below the optimal level, could also occur in some natural soils.

Suboptimal nitrate concentrations may cause stress to plants, and acute stresses can either delay or induce flowering (Kazan and Lyons, 2016; Takeno, 2016). To investigate this further, the expression of several stress-related marker genes, including *RD29A*, *RD29B*, *KIN1*, and *PAP1*, were determined (Supplementary Figure 5). Under both LD and SD conditions, the expression of these genes was significantly decreased when the nitrate supply was increased from 0.2 mM to 1.0 mM, indicating that the promotion of flowering by increase of nitrate supply below 1.0 mM may be associated with the alleviation of nitrogen-deficiency stress. Increasing the nitrate supply above 1 mM, however, had little effect on the transcript levels of these stress-related marker genes, except for *KIN1* in 16-mM nitrate treatment (Supplementary Figure 5). Considering that the plants still flowered earlier in the 2-mM nitrate treatment than in the 1-mM nitrate treatment, under both LD and SD conditions (Figure 1 and Supplementary Figures 1, 3), the floral induction from increasing the suboptimal nitrate supply, may not be solely associated with the alleviation of nitrogen-deficiency stress.

### LD Induction of Flowering Is Associated With Nitrate Availability

**FIGURE 2:**
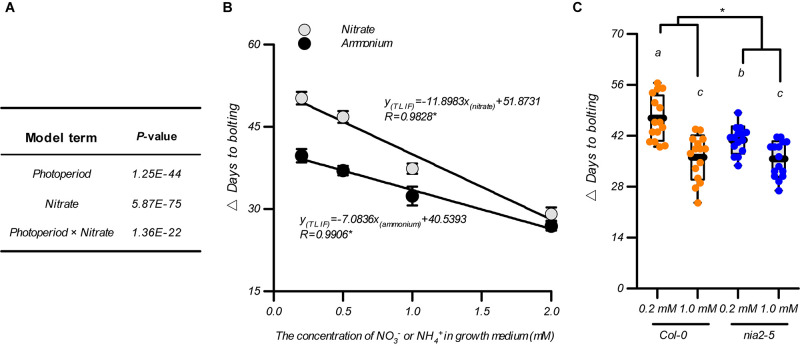
Association of flowering under LD conditions with nitrate availability. **(A)** Statistical analysis of the data from Figures 1A,C. Data were analyzed using a two-way ANOVA followed by Duncan’s multiple range test. **(B)** The time of LD-induced flowering (△ days to bolting) in the Col-0 plants grown with different nitrogen sources. Different concentrations of (NH_4_)_2_SO_4_ including 0.2, 0.5, 1.0, and 2.0 mM were used to replace the KNO_3_ in the agar medium. **(C)** The time of LD-induced flowering (△ days to flowering) in the Col-0 plants and *nia2-5* mutants, grown with 0.2 mM or 1 mM nitrate. Seedlings were grown on agar media containing nitrate or ammonium as indicated. The potassium concentrations in all growth media were adjusted to 1 mM using K_2_SO_4_. LD: 16-h light/8-h dark; SD: 8-h light/16-h dark. △ Days to bolting was calculated by subtracting the bolting time of plants grown under LD conditions from that of plants grown under SD conditions, with the same nitrogen treatment. Raw data of panels **(B,C)** from individual photoperiods are shown in Supplementary Figures 6, 7. At least 20 plants grown in each of the LD and SD conditions were used for the quantification of flowering. Error bars represent ± standard deviations. Different letters indicate significant differences between means, as determined using a two-way ANOVA followed by Tukey’s multiple comparisons test (*P* < 0.05). Asterisk indicates significant interaction of photoperiod × nitrogen source **(B)** or photoperiod × genotype **(C)** at *P* < 0.05 (two-way ANOVA).

As flowering is promoted by the elevation in nitrate availability below an optimal level under both LD and SD conditions, we investigated whether the nitrate availability in the growth medium affected the photoperiodic flowering response. Two-way ANOVAs are reported as an effective way to test the interactions between these two treatments (Brady et al., 2015) and were consequently used for data analysis as shown in Figures 1A,C. In addition to the unilateral impact of the photoperiod or nitrate availability, a significant interaction between these two environmental factors was also observed in the control of the flowering time of the plants, that is, the *P*-value of the model term “photoperiod × nitrate” was 1.36E^–22^ (Figure 2A). Therefore, we investigated how nitrate availability affects the photoperiodic flowering responses in plants. By subtracting the bolting time of the plants grown under LD conditions from those grown under SD conditions, within the same nitrogen treatment, a time interval for bolting (△ days to bolting), defined as the time of LD-induced flowering (TLIF), was generated. The results showed that the increases in the nitrate supply clearly minimized the TLIF (e.g., TLIF = 50.2 d in 0.2-mM nitrate treatment and 29.1 d in 2-mM nitrate treatment). These results indicated that flowering under SD conditions could partially mimic the flowering under LD conditions if the nitrate availability was improved. We also fitted the TLIF and the level of nitrate supply data with a linear model and found that the TLIF was negatively correlated with the level of nitrate supply (*R* = 0.9828, Figure 2B), thus providing further support for the above conclusions.

In addition to being an essential nutrient (i.e., nitrogen source) for plant growth and development, nitrate also serves as a signaling molecule that regulates several physiological processes (Alboresi et al., 2005; Konishi and Yanagisawa, 2013; Krapp et al., 2014; Bouguyon et al., 2015; Maeda et al., 2018; Olas et al., 2019). To clarify how nitrate functions as a nitrogen source to control photoperiod-modulated flowering, we tested the TLIF of Col-0 plants grown with ammonium as an alternative N source (the individual LD and SD data sets are shown in Supplementary Figure 6). The results showed that the TLIF was linearly correlated when the level of ammonium supplied was ranged from 0.2 to 2 mM (*R* = 0.9906 Figure 2B), indicating that the nitrogen-source action of the nitrate may be a part of the nitrate mechanism for controlling the TLIF. However, when comparing this TLIF-ammonium regression line with the above TLIF-nitrate regression line, using a UNIANOVA, we found that the decrease in the TLIF in response to the elevated nitrate supply was significantly steeper than in response to the elevated ammonium supply. This difference could also be seen by comparing the slopes of the above two regression lines (-11.890 and -5.67 for TLIF-nitrate and TLIF-ammonium regression lines, respectively) (Figure 2B). We then analysis the flowering of *nia2-5* mutant, which has less than 10% of the wild-type nitrate reductase activity (Desikan et al., 2002; Wang et al., 2004; Seligman et al., 2008), was used for further investigations. As shown in Figure 2C, in comparison with that in the Col-0 plants, the difference in TLIF between the 0.2 mM and 1 mM nitrate treatments was clearly diminished in the *nia2-5* mutants, suggesting that the sensitivity of the photoperiodic flowering responses to elevated levels of nitrate were also reduced by the dysfunction of the nitrate assimilation pathway. Moreover, the increase in nitrate supply still led to a statistically significant decrease in TLIF in the *nia2-5* mutants (Figure 2C and Supplementary Figure 7). Collectively, these results indicated that in addition to the nitrate’s nutritional function, it may also function as a signaling-molecule to induce flowering during extended photoperiods.

### LD Photoperiods Upregulate Nitrate Uptake by NRT1.1/NRT2.1

**FIGURE 3:**
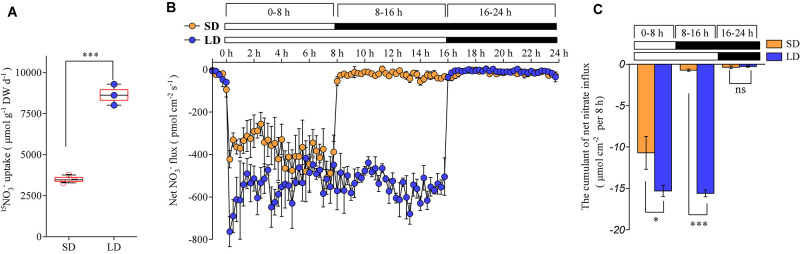
LD photoperiod treatment increases the root nitrate uptake. Nitrate (1 mM) was used as the exclusive N source in this experiment. **(A)** Daily nitrate uptake of the roots in LD (16-h light/8-h dark) or SD (8-h light/16-h dark) conditions. Col-0 plants were grown hydroponically for 2 weeks before being harvested for the measurement of ^15^NO_3_^–^ uptake. Error bars represent ± standard deviations (*n* = 3) of four biological replicates. **(B)** Dynamic changes in the net NO_3_^–^ flux in the roots of Col-0 plants grown under LD or SD conditions. Bars above the graph indicate the duration of day (white) and night (black). Seedlings (at the stage when four leaves were visible) grown in agar medium were used to measure the net nitrate fluxes in the maturation zones. Each data point represents the average of the net nitrate flux over 15 min. Negative symbols indicate net influx; error bars represent means ± standard deviations (*n* = 4). **(C)** The cumulated net nitrate influx calculated for each 8-h period in the roots of the Col-0 plants grown under the LD or SD conditions. The value of each period (0–8 h, 8–16 h, or 16–24 h) was calculated according to the corresponding period in **(B)**. Asterisks indicate significant differences between treatments (^∗^*P* < 0.05, ^∗∗∗^*P* < 0.001; one-way ANOVA with Tukey’s multiple comparisons test).

We investigated how the photoperiod affects root nitrate uptake in plants. The daily nitrate uptake rates by the roots of the Col-0 plants were compared between the LD and SD treatments. The plants were hydroponically pre-cultured with an intermediate concentration of nitrate (1 mM), under either LD or SD conditions. The daily nitrate uptake rates by the roots were then evaluated using ^15^NO_3_^–^ under the same conditions used for the plant growth. The results showed that they increased by about 1.6-fold with the LD treatment when compared to the SD treatment (Figure 3A). The non-invasive micro-test technique, which uses computer-controlled stepper motors to oscillate an ion-selective microelectrode near the surfaces of the tissues, could provide a real-time measurement of the ion flux based on Fick’s first law of diffusion (Shabala et al., 2016). Therefore, this technique was used to continuously monitor the transmembrane NO_3_^–^ fluxes in the roots over a 24-h period. The maturation zone in roots has a greater net nitrate influx than the other root zones and this root zone makes up most of the root surface area (Zhu et al., 2019). Therefore, the transmembrane NO_3_^–^ fluxes of the roots in the maturation zones were monitored in this study, to investigate the effects of photoperiod on root nitrate uptake. Although the net nitrate influx in the roots was observed under dark conditions, it was extremely low with both LD and SD treatments (Figures 3B,C). In accordance with earlier studies (Delhon et al., 1995; Peuke and Jeschke, 1998), the net nitrate influx of the roots was greatly increased under the light conditions, when compared with the dark conditions (Figures 3B,C). Interestingly, the LD treatments resulted in a higher net nitrate influx than the SD treatments, despite the plants being illuminated during the first 8 h per day under both photoperiod conditions (Figure 3B). In addition, the cumulant calculation showed that the net nitrate influx increased by ∼35% with the LD treatment, when compared with the SD treatment (Figure 3C). These results suggest that both a longer illumination time and a higher nitrate uptake rate during the illumination period contributed to the improved nitrate uptake under LD conditions.

**FIGURE 4:**
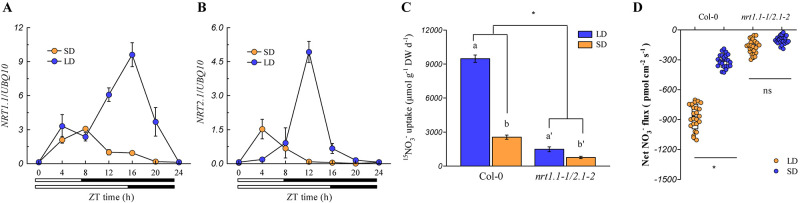
LD photoperiods upregulate NRT1.1/NRT2.1*-*mediated nitrate uptake in the roots. The expression pattern of *NRT1.1* **(A)** and *NRT2.1* **(B)** in the roots of Col-0 plants grown under LD (16-h light/8-h dark) and SD (8-h light/16-h dark) photoperiods. Seedlings (at the stage when four leaves were visible) grown in agar medium with 1 mM KNO_3_ were harvested. Bars below the graph indicate the duration of day (white) and night (black). Expression levels are normalized to the expression level of *UBIQUITIN 10*. **(C)** Nitrate uptake rate in the roots of the Col-0 plants and *nrt1.1-1/2.1-2* mutants under both LD and SD conditions. The plants were grown hydroponically for 2 weeks with 3 mM KNO_3_ to ensure that the *nrt1.1-1/2.1-2* plants would not show severe signs of N limitations. The plants were then grown in 1 mM nitrate for another week, after which the daily nitrate uptake rate by the roots was evaluated using 1 mM ^15^NO_3_^–^. **(D)** Supplementary Figure 14 Net NO_3_^–^ flux in the roots of Col-0 plants and *nrt1.1-1/2.1-2* mutants grown in agar systems under SD and LD conditions. Nitrate (1 mM) was used as the exclusive N-source in this section. Seedlings (at the stage when four leaves were visible) grown in agar medium were used to measure the net nitrate fluxes in the maturation zones at ZT 1-2 h. Data represent the means ± standard deviations (*n* = 4). Different letters indicate significant differences between the means as determined by using a two-way ANOVA followed by Tukey’s multiple comparisons test (*P* < 0.05). Significant interactions between the photoperiod and genotype are indicated by an asterisk (*P < 0.05; ns, non-significant).

Currently, six nitrate transporters (NRTs) have been characterized to be involved in root nitrate uptake in *Arabidopsis* plants. Among these, NRT1.1 and NRT2.1 play the most pivotal roles in root nitrate uptake in most growth environments (Li et al., 2006), whereas NRT1.2, NRT2.2, NRT2.4, and NRT2.5 only contribute to minor levels of root nitrate uptake (Wang et al., 2012; Lezhneva et al., 2014). The expression of *NRT1.1* and *NRT2.1* in the roots was analyzed to understand how these two NRTs act to control root nitrate uptake under different photoperiod conditions. As shown in Figures 4A,B, these two genes were markedly upregulated during the day-time in both LD- and SD-grown plants, but the up-regulation of both genes was much higher at the peak time point in the LD-grown plants (ZT 16 h for *NRT1.1* and ZT 12 h for *NRT2.1*) than in the SD-grown plants (ZT 8 h for *NRT1.1* and ZT 4 h for *NRT2.1*). As shown in Supplementary Figure 8, *NRT2.2*, *NRT2.4*, and *NRT2.5* were differentially regulated by the photoperiod, and their expression was much lower than that of the *NRT1.1* and *NRT2.1* (Figure 4). In addition, the expression of *NRT1.2* was barely affected by the photoperiod. These results, together with the fact that NRT1.1 and NRT2.1 are crucial for root nitrate uptake under most conditions (Li et al., 2006; Wang et al., 2012; Lezhneva et al., 2014), indicated that higher expression levels of *NRT1.1* and *NRT2.1* in the roots of the LD-grown plants may be the most important factor leading to the higher root nitrate uptake when compared with the SD-grown plants. The *nrt1.1-1/nrt2.1-2* double mutant, which possessed T-DNA insertions in the exon and intron of the *NRT1.1* and *NRT2.1* genes, respectively, were then used to clarify the above speculations. Although the T-DNA insertion in the intron of the *NRT2.1* gene also leads to the deletion of its adjacent gene *NRT2.2* (Li et al., 2006; Ye et al., 2019), the effect of this deletion on the root nitrate uptake could be limited in our present growth conditions for the *nrt1.1-1/nrt2.1-2* double mutant, because the contribution of *NRT2.2* to the root nitrate uptake was very small (Li et al., 2006). In this study, we found that the loss of action of NRT1.1/NRT2.1 in the *nrt1.1-1/nrt2.1-2* mutants substantially inhibited the daily nitrate uptake rate (Figures 4C,D). Furthermore, the two-way ANOVA analysis revealed that the LD increased the daily nitrate uptake rate in the *nrt1.1-1/nrt2.1-2* mutants, which was much lower than that in the Col-0 plants. Collectively, these results suggested that the LD-improved nitrate uptake by roots may be associated with the up-regulation of *NRT1.1* and *NRT2.1.*

### LD-Induced Flowering Is Associated With the Action of *NRT1.1/NRT2.1*

**FIGURE 5:**
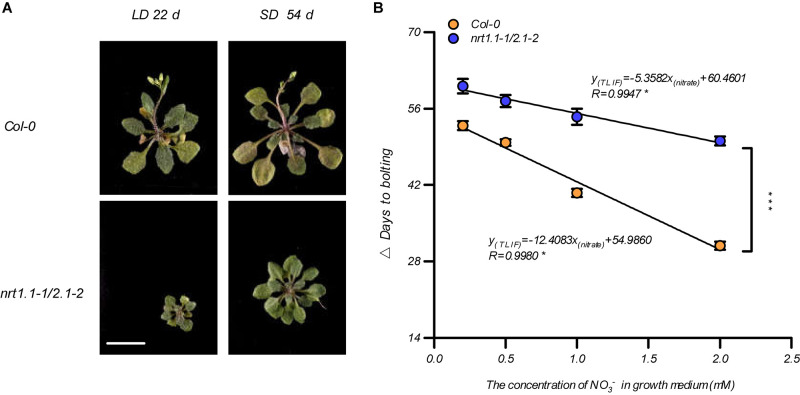
NRT1.1- and NRT2.1-mediated nitrate uptake confers LD-induced flowering. **(A)** Phenotypic photographs of Col-0 and *nrt1.1-1/2.1-2* plants treated with 1.0 mM nitrate. The plants were grown in an agar system containing nitrate as the sole N source, under SD and LD conditions. Scale bar = 1 cm. **(B)** The time of the LD-induced flowering (Δ days to bolting) in the Col-0 plants and *nrt1.1-1/2.1-2* mutants. Seedlings were grown on agar medium containing a series of KNO_3_ concentrations, as indicated. The potassium concentrations in all growth media were adjusted to 2 mM using K_2_SO_4_. SD: 8-h light/16-h dark; LD: 16-h light/8-h dark. Δ Days to bolting was calculated by subtracting the bolting time of the plants grown under LD conditions from that of the plants grown under SD conditions, with the same nitrogen treatment. Raw data from individual photoperiods are shown in Supplementary Figure 10. At least 20 plants grown in each of the LD and SD conditions were used for the quantification of flowering. Error bars represent ± standard deviations (*n* = 12). The regression lines were compared using a UNIANOVA. Significant differences between two regression lines were indicated by an asterisk (***P < 0.0001).

Given that the LD conditions improved the root nitrate uptake by affecting the NRT1.1 and NRT2.1, we aimed to clarify our previous speculation that photoperiodic flowering responses may be associated with alternative nitrate uptake. We first compared the bolting time of the *nrt1.1-1* mutants, the *nrt2.1-2* mutants, and the *nrt1.1-1/2.1-2* mutants in a soil system. As shown in Supplementary Figure 9, both *nrt1.1-1* and the *nrt2.1-2* bolted later than Col-0, but earlier than the *nrt1.1-1/2.1-2* mutants. Furthermore, the *nrt1.1-1/2.1-2* mutants had a higher TLIF than that of *nrt1.1-1* and *nrt2.1-2*. This result indicated that NRT1.1 and NRT2.1 were additively involved in LD-induced flowering. Therefore, the flowering responses of the Col-0 plants and the *nrt1.1-1/nrt2.1-2* mutants were further compared using different photoperiod treatments in the agar medium, with 1 mM nitrate. Figure 5A shows that the main inflorescence of the Col-0 plants reached a height of approximately 2 cm on the 22nd and 54th day of growth under the LD and SD treatments, respectively, but *nrt1.1-1/nrt2.1-2* mutants had not yet bolted in both treatments. We then analyzed the TLIF of the Col-0 plants and *nrt1.1-1/2.1-2* mutants in the media with different nitrate supplies (the individual LD and SD data sets are shown in Supplementary Figure 10). Although the TLIF of the *nrt1.1-1/nrt2.1-2* mutants was also linearly and negatively correlated with nitrate concentrations below 2 mM (*R* = 0.9947, Figure 5B), the slope of the regression line was significantly smaller than that of the Col-0 plants (UNIANOVA, *P*-value < 0.0001), which suggested that the loss of action of the NRT1.1/NRT2.1 in the *nrt1.1-1/2.1-2* mutants reduced the sensitivity of the photoperiodic flowering responses to the elevated levels of nitrate. Moreover, the TLIF in the *nrt1.1-1/2.1-2* mutants, which mimicked the TLIF of the Col-0 plants in the lower nitrate treatments, was significantly increased, compared with that of the wild-type plants with the same nitrate concentrations. Collectively, these results indicate that the actions of the NRT1.1/NRT2.1 were involved in the LD-induced flowering.

**FIGURE 6:**
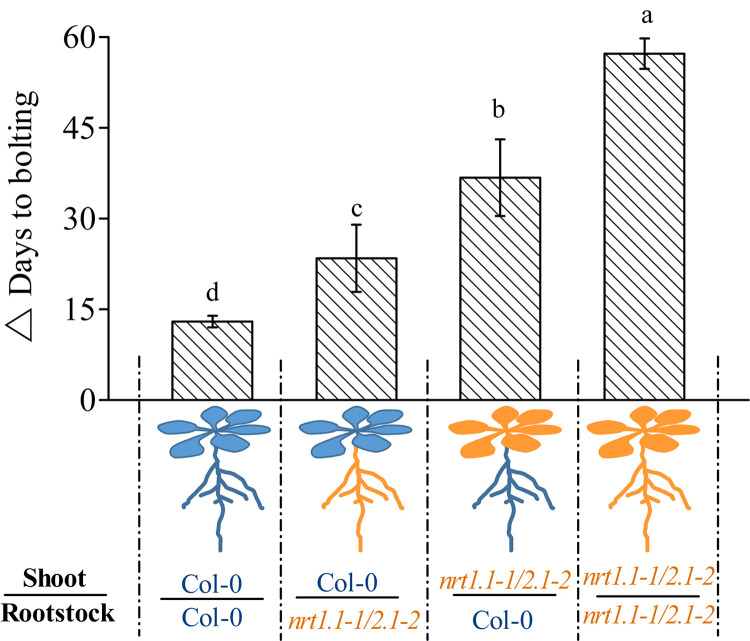
Time of LD-induced flowering in grafted plants under 1 mM nitrate conditions. △ Days to bolting was determined by subtracting the bolting time of the plants grown under LD conditions from that of the plants grown under SD conditions. Raw data from the individual photoperiods are shown in Supplementary Figure 11. Treatments are similar to those presented in Figure 3. Bars represent means ± standard deviations (*n* = 6). Different letters above the bars indicate significant differences between the means, as determined using a one-way ANOVA followed by Tukey’s multiple comparisons test (*P* < 0.05).

Given that *NRT1.1* and *NRT2.1* are also expressed in the shoots (Guo et al., 2001; Okamoto et al., 2003), the role of NRT1.1/NRT2.1 is clearly not limited to nitrate uptake in the roots. We aimed to determine whether *NRT1.1*/*NRT2.1* only confers LD-induced flowering by affecting nitrate uptake in the roots or by also affecting it in their shoot-part functions. Therefore, we reciprocally grafted *nrt1.1-1/2.1-2* shoot scions onto wild-type rootstocks (*nrt1.1/2.1*/Col-0) and wild-type shoot scions onto *nrt1.1-1/2.1-2* rootstocks (Col-0/*nrt1.1/2.1*), and determined the flowering time of the grafted combinations. Plants were also homo-grafted as controls (Col-0 scion/Col-0 stock and *nrt1.1-1/2.1-2* scion/*nrt1.1-1/2.1-2* stock). As shown in Figure 6, the TLIF of the *nrt1.1-1/2.1-2* rootstocks grafted with the Col-0 shoots (Col-0/*nrt1.1/2.1*) was significantly higher than that of the self-grafted wild-type plants (the individual LD and SD data sets are shown in Supplementary Figure 11). Given the above finding that the LD conditions upregulated the nitrate uptake of the NRT1.1/NRT2.1, these results suggested that root nitrate uptake was controlled by NRT1.1/NRT2.1 and should be involved in LD-induced flowering. This assumption was further supported by the observation that TLIF was significantly decreased in the shoots of *nrt1.1-1/2.1-2* grafted with the Col-0 rootstocks.

Interestingly, we also found that the TLIF of the *nrt1.1/2.1*/Col-0 grafts was significantly increased compared with that of the Col-0/Col-0 grafts. These results suggest that the shoot-part function of the NRT1.1/NRT2.1 was also involved in the LD-induced flowering. It is noteworthy that the TLIF of the *nrt1.1/2.1*/Col-0 grafts was even greater than that of the Col-0/*nrt1.1/2.1* grafts, indicating that the shoot-part function of the NRT1.1/NRT2.1 may contribute more to the LD-induced flowering than the root-part functions.

### Improved Nitrate Status in the LD-Induced Flowering Was Associated With the Up-Regulation of Flowering-Related Genes

**FIGURE 7:**
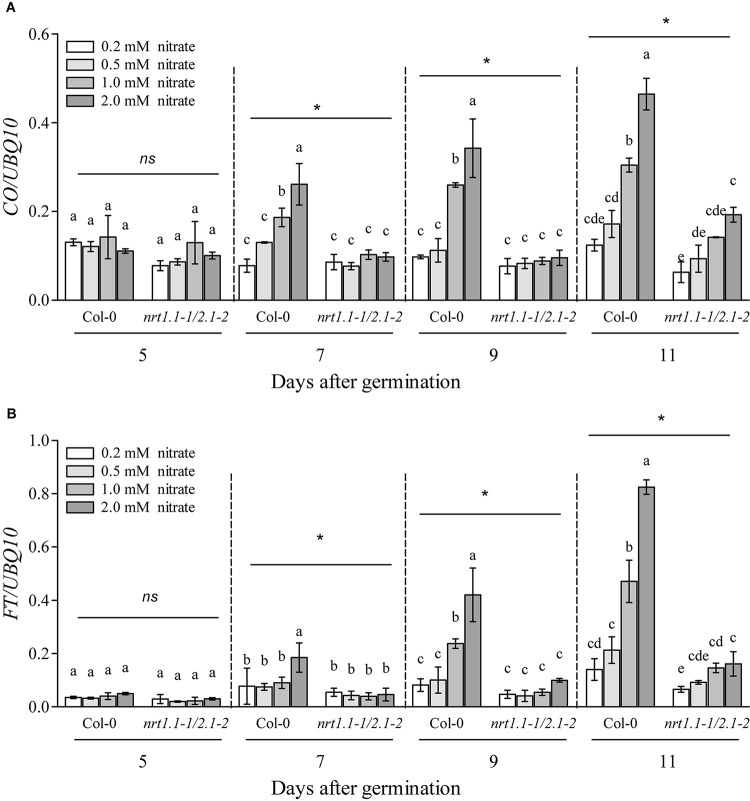
Loss of function of NRT1.1 and NRT2.1 suppressed the induction of *FT* and *CO* by increasing nitrate supplies under LD conditions. Temporal expression of *CO* **(A)** and *FT* **(B)** in the shoots of Col-0 and *nrt1.1-1/2.1-2* plants grown in 0.2, 0.5, 1.0, and 2.0 mM nitrate treatments, under LD conditions. Plants were harvested at zeitgeber time (ZT) 16 h for RNA extraction, after the indicated number of germination days. Potassium concentrations in all growth media were adjusted to 2 mM using K_2_SO_4_. Expression levels are normalized to the expression level of *UBIQUITIN 10*. Bars represent means ± standard deviations (*n* = 4). Different letters indicate significant differences between the means, as determined using a two-way ANOVA followed by Tukey’s multiple comparisons test (*P* < 0.05). Significant interactions between photoperiod and genotype are indicated by an asterisk (*P < 0.05; ns, non-significant).

**FIGURE 8:**
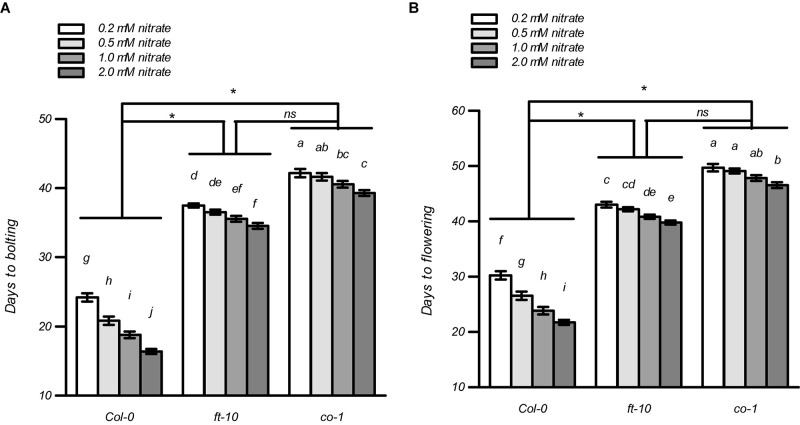
Effects of nitrate availability on flowering in *ft-10* and *co-1* mutants under LD conditions. Days to bolting **(A)** and days to flowering **(B)** of the plants grown with different nitrate concentrations, were determined in an agar system under LD conditions (16 h light/8 h dark). Bars represent means ± standard deviations (*n* = 20). Different letters indicate significant differences between the means, as determined using a two-way ANOVA followed by Tukey’s multiple comparisons test (*P* < 0.05). Significant interactions between the photoperiod and genotype are indicated by an asterisk (*P < 0.05; ns, non-significant).

In most cases, flowering induced by the LD photoperiod was under the control of the canonical genetic GI-CO-FT pathway (Fornara et al., 2010; Tiwari et al., 2010; Andrés and Coupland, 2012; Yuan et al., 2016; Luo et al., 2018; Bao et al., 2019). Therefore, we first investigated whether the nitrate availability at different concentrations affected the expression of several key genes involved in this pathway, including *GI*, *FKF1*, *COP1*, *CO*, and *FT*. The plants at the four-leaf visible stage were used to investigate gene expression. As shown in Supplementary Figure 12, only the expression of *CO* and *FT* were upregulated dramatically in the Col-0 plants with 1 mM nitrate when compared to the 0.2-mM nitrate treatments under LD conditions; in contrast, the loss of function of NRT1.1/NRT2.1 in the *nrt1.1-1/2.1-2* mutants strongly inhibited this increase. We then investigated the temporal expression (5, 7, 9, and 11 days after germination [DAG]) of *FT* and *CO* in the shoots of the seedlings treated with the different nitrate concentrations under LD conditions (Figure 7). The results showed that at 5 DAG, the expression of both genes was not significantly different from the different nitrate supplies. However, the elevation in nitrate supply increased the expression of the *CO* and *FT* from 7 and 9 DAG, respectively. Similar to the results observed above, the differences in the expression of these two genes was clearly reduced by the loss of function of NRT1.1/NRT2.1, indicating that the induction of *CO* and *FT* with the LD photoperiod was associated with an improved nitrate status in plants. Subsequently, we compared the flowering responses between the Col-0 plants and the *co* and *ft* mutants under LD conditions. As shown in Figure 8 and Supplementary Table 2, the elevation in the nitrate levels had a smaller effect on the promotion of flowering in both *co-1* and *ft-10* mutants, when compared with the Col-0 plants. Taken together, the contributions of the improved nitrate status to the LD photoperiod-induced flowering is partially associated with the upregulation of *CO* and *FT* expression.

In addition to the CO-FT pathway, Yuan et al. (2016) indicated that the blue-light receptor cryptochrome 1 (CRY1) also played a critical role in the N-regulated flowering of *Arabidopsis*. Considering that the elevation in nitrate supply were found to increase *CRY1* expression (Supplementary Figure 12G), the contribution of the improved nitrate status to the up-regulation of *CRY1* may also play a role in LD-induced flowering. Therefore, we also determined the effects of nitrate availability below the optimal level for the flowering of CRY1 loss-of-function mutants under both SD and LD conditions (Supplementary Table 3). The results showed that increasing the nitrate concentration had no significant effect on the promotion of flowering in the *cry1* mutants, under either LD or SD conditions, supporting the above assumption. Interestingly, the elevation in nitrate concentrations also increased the number of rosette leaves in the *cry1* mutants, but this increase was less than that observed in the Col-0 plants (Supplementary Table 3). Therefore, the process by which nitrate affects plant vegetative growth might be partially associated with CRY1.

## Discussion

To ensure reproductive success and seed production, the flowering of plants is controlled by sophisticated regulatory networks that monitor changes in the environment, especially changes in the photoperiod (Andrés and Coupland, 2012; Yuan et al., 2016; Luo et al., 2018; Bao et al., 2019, 2020). In this study, we have reported that nitrate play a role in LD-induced flowering. We found that when the nitrate availability in the growth system was below the optimal level for the flowering of plants, the LD conditions improved the nitrate status by upregulating NRT1.1/NRT2.1 expression, which subsequently upregulates the expression of light-responsive regulators *FT* and *CO* in plants, thereby modulating the timing of floral induction.

As demonstrated previously, and in this study, the elevation in nitrate availability in the growth system below an optimal level resulted in a clear acceleration of the flowering process (Lin and Tsay, 2017; Figure 1 and Supplementary Figure 1), which decreased the difference in flowering time between the LD and SD conditions (Figure 2 and Supplementary Figure 3). This suggested that the photoperiodic flowering response is associated with the nitrate status in Arabidopsis plants, and that flowering under LD conditions could be partially mimicked under SD conditions if nitrate concentrations were elevated. As an essential nutrient for plants, nitrate can greatly affect plant growth and development, such as seed germination, shoot branching, lateral root formation, and flowering (Alboresi et al., 2005; Castro Marín et al., 2011; Bouguyon et al., 2015; Zeng et al., 2017). Previous studies have indicated that the number of rosette leaves at flowering is related to the days to flowering under most conditions when there is a sufficient supply of nitrate (Koornneef et al., 1991; Bagnall, 1993; Pouteau et al., 2004; Pouteau and Albertini, 2009; Lin and Tsay, 2017; Gras et al., 2018). Therefore, the number of rosette leaves at flowering is often used as an indicator of floral transition under most conditions when there is a sufficient supply of nitrate. However, in an agar-based growth system with suboptimal nitrate concentrations, we found that the flowering time was negatively correlated with the number of rosette leaves and biomass production under both LD and SD conditions (Figure 1 and Supplementary Figures 1, 10). Of note, in a soil-based growth system, Lin and Tsay (2017) also found that plants grown in 1.0 mM nitrate had shorter flowering times and more rosette leaves than plants grown in 0.4 mM nitrate. We assumed that the floral transitions below the optimal nitrate concentrations may not merely be a consequence of the cessation of leaf production, and that other factors affecting the quantitative responses might also be involved. The shortage of nitrogen for leaf production under low nitrate conditions may explain the aforementioned results. However, more studies are required to draw clear conclusions. We also found that the acceleration in flowering observed with the elevation in nitrate availability, below the optimal level, could also occur in some natural soils (Supplementary Figure 4). However, the optimal nitrate level for promoting flowering in the soil system is higher than that in the agar system (Supplementary Figures 3, 4). Therefore, although the results observed in the agar system could help predict results in the soil system, caution should be exercised when directly applying these results in natural systems. Notably, the promotion of flowering and the increase in the number of rosette leaves resulting from the elevation in nitrate concentrations under SD conditions were more significant than those under LD conditions (Supplementary Figure 3). A similar result was also observed in the investigation by Castro Marín et al. (2011). This is probably because growth is much slower and the vegetative period is extended under SD conditions that the effect of nitrate availability on plant vegetative growth becomes more prominent. Furthermore, although nitrate at high concentrations (>4 mM) did not alter flowering time, nitrate below the optimal level did promote plant growth and the flowering of plants under LD conditions (Figure 1 and Supplementary Figures 2, 3), suggesting that a faster rate of growth under LD conditions may mask the effect of excessive nitrate on plant growth and flowering. Therefore, the role of nitrate as a nutrient may elucidate the mechanism involved in LD-induced flowering. This notion was further confirmed by the observation that the elevation in ammonium levels, which is an intermediate product of nitrate assimilation, also minimized TLIF (Figure 2B and Supplementary Figure 6). A plausible explanation may be that the role of nitrate as a nutrient source may also act to control the strong nitrate response of SD-dependent flowering.

Besides its role as a nitrogen source, nitrate is also functional in signal-regulated gene expression, metabolism, growth, and development processes in plants (Wang et al., 2012; Bouguyon et al., 2015). Very recently, Olas et al. (2019) observed that flowering was markedly delayed in *Arabidopsis nlp6*/*nlp7* double mutants, as there were disruptions in two master regulators of nitrate signaling, demonstrating the vital role of nitrate signaling in regulating the flowering of plants. In this study, we showed that LD-induced flowering may also be associated with the non-nutritional function of nitrate. This was supported by the finding that the sensitivity of the photoperiodic flowering responses to elevated levels of nitrate was reduced by either the replacement of nitrate with ammonium (Figure 2B and Supplementary Figure 6) or the dysfunction of the nitrate assimilation pathway (Figure 2C and Supplementary Figure 7). In addition, Olas et al. (2019) identified the shoot apical meristem as important site for nitrate signal perception to induce flowering. Our findings that the shoot functions of the NRT1.1 and NRT2.1 were also involved in LD-induced flowering (Figure 6 and Supplementary Figure 11), may also be associated with the role of nitrate in the SAM for floral induction during extended photoperiods. Previous studies have shown that *NRT1.1* and *NRT2.1* are also expressed in shoots (Guo et al., 2001; Okamoto et al., 2003), indicating that the role of NRT1.1 and NRT2.1 is not limited to nitrate uptake in the roots. As the fundamental functions of both NRT1.1 and NRT2.1 are for nitrate transport across the plasmalemma, it may be a plausible explanation that the NRT1.1 and NRT2.1 in shoots are also involved in distributing nitrate to different shoot tissues. In addition, NRT1.1 is also recognized as a nitrate sensor and NRT2.1 is a putative nitrate sensor (Remans et al., 2006; Ho et al., 2009). The NRT1.1/NRT2.1 in the shoots may also act to transmit nitrate signals to promote flowering. Furthermore, nitrate assimilation may also play a role in the shoot-part function of NRT1.1/NRT2.1 in regulating LD-induced flowering because the distribution of nitrate in different shoot tissues owing to NRT1.1/NRT2.1 could indirectly affect nitrate assimilation. Future studies are required to clarify how the NRT1.1 and NRT2.1 in SAM are involved in LD-induced flowering.

As previously mentioned, the floral responses to photoperiod alterations and some environmental cues, such as UV light, cold, and salt, are in most cases, under the control of the canonical genetic CO-FT pathway (Guo and Yang, 1998; Martínez et al., 2004; Fornara et al., 2010; Tiwari et al., 2010; Andrés and Coupland, 2012; Luo et al., 2018). FT is a florigen signal that is first expressed in the leaf but induces floral initiation in the SAM (Andrés and Coupland, 2012; Luo et al., 2018). The movement of FT protein through the phloem to the apex has been confirmed in many species (Corbesier et al., 2007; Lin et al., 2007; Aki et al., 2008). CO is another key regulator that encodes a nuclear protein and positively regulates the flowering of plants by increasing FT expression (Putterill et al., 1995). Briefly, light promotes the accumulation of CO. The CO then interacts with the B and C subunits of nuclear factor Y (NF-Y), to form the NF-CO complex, which is bound to the proximal region of the *FT* promoter and acts to increase *FT* expression (Tiwari et al., 2010; Andrés and Coupland, 2012; Luo et al., 2018). Very recently, Teng et al. (2019) showed that NRT1.1 regulates flowering in an FT-dependent but CO-independent manner. It is worth noting that a disruption in *NRT1.1* induced the expression of *NRT2.1*, and thus the nitrate uptake of the *nrt1.1* mutant was significantly improved under nitrate-limited conditions (Muños et al., 2004; Bouguyon et al., 2015; Ye et al., 2019). The induction of *NRT2.1* may interfere with the regulation of NRT1.1 for *CO* expression, under suboptimal nitrate conditions. Therefore, in this study, we observed that the LD-induced expressions of *CO* and *FT* were both decreased by the dysfunction of the nitrate transporters NRT1.1 and NRT2.1 (Figure 7). These results indicate that the action of nitrate in LD-induced flowering probably depends on the up-regulation of the CO-FT pathway. This notion was supported by the observation that the flowering that was induced by increasing the nitrate levels was restrained by the loss of function of either CO or FT under LD conditions (Figure 8). The link between nitrate availability and *FT* expression was also indicated in a previous study by Gras et al. (2018). They found that the mRNA expression of *FT* peaked when plants were grown in optimal nitrate concentrations, but not at higher concentrations. In addition, they showed that the flowering time of *ft-10* did not respond to the nitrate supply above the optimal nitrate level. Moreover, nitrate-controlled *FT* expression was associated with the floral repressors SMZ and SNZ, as the early accumulation of these repressors in high nitrate concentrations repressed FT induction and delayed flowering. However, in this study, we observed that increasing the nitrate concentrations, below the optimal level, still led to earlier flowering in both *co-1* and *ft-10* mutants under LD conditions; although this induction was largely lower than that in the Col-0 plants (Figure 8). These results suggested that the up-regulation of *CO* and *FT* by nitrate has a role in promoting flowering under LD conditions.

Although several NRTs have been characterized to be involved in root nitrate uptake in *Arabidopsis* plants, NRT1.1 and NRT2.1 contribute to the majority of root nitrate uptake under both nitrate-limited and nitrate-sufficient conditions (Li et al., 2006; Wang et al., 2012; Ye et al., 2019). Interestingly, the LD conditions clearly increased the root nitrate uptake by the up-regulation of *NRT1.1* and *NRT2.1* expression (Figures 3, 4). This led to speculation as to how the LD conditions increased the nitrate uptake using NRT1.1 and NRT2.1. It is widely acknowledged that carbon photo-assimilates are vital signals involved in nitrate uptake, and that extended photoperiod conditions elevate the accumulation of carbon photo-assimilates (Nunes-Nesi et al., 2010; Chen et al., 2016). This suggests that the LD-improved root nitrate uptake may be associated with the increased production of photosynthates in plants. This notion was supported by the finding that the exogenous application of sucrose, a typical photo-assimilate, clearly upregulated the expression of *NRT1.1* and *NRT2.1*, and improved root nitrate uptake in dark-grown *Arabidopsis* (Lejay et al., 1999, 2003). In contrast, direct regulation of light-responsive transcription factors on nitrate uptake genes may also be an alternative mechanism underlying the improved LD nitrate uptake of the roots. The finding that the light-responsive transcription factor HY5 can directly bind to the promoter of *NRT2.1* to improve the expression of *NRT2.1*, provides evidence to support this speculation (Chen et al., 2016). In addition, three putative binding sites for HY5 have also been identified in the promoter region of *NRT1.1*, which underpinned the hypothesis that the regulation of HY5 on the expression of *NRT1.1*, may involve direct binding to the *NRT1.1* promoter (Lee et al., 2007; Jonassen et al., 2009).

In summary, improved nitrate status plays a role in LD-induced flowering in *Arabidopsis* plants. To understand if the nitrate status related to the LD-induced flowering in *Arabidopsis* plants could be generalized to other plants, multiple species need to be tested in a range of ecosystems. As previously mentioned, plants usually face N insufficiencies in most ecosystems; however, the global emission and deposition levels of fixed atmospheric nitrogen have been markedly enhanced over the past 70 years because of the development of intensive agriculture and the combustion of fossil fuels (Galloway, 1995; Stevens et al., 2004). If the photoperiodic flowering responses in other plants can also be adjusted by altering the nitrate supply, our results may provide a physiological basis for the effects of N depositions on plant reproductive dynamics and enable improved predictions of the future changes in the natural reproductive rhythms of plant populations in most terrestrial ecosystems. In addition, our findings may also help to develop more accurate nitrate management protocols to improve crop performance, by controlling the timing of flowering in crops.

## Data Availability Statement

The original contributions presented in the study are included in the article/Supplementary Material, further inquiries can be directed to the corresponding author.

## Author Contributions

JY, WT, and CJ executed the experiments, interpreted the data, and generated the figures. MZ assisted in generating *nrt1.1-1*/*nrt2.1-1* mutants and performing the grafted experiment. QZ and WD assisted in the analysis of data. JY and CJ wrote the manuscript. All authors contributed to the article and approved the submitted version.

## Conflict of Interest

The authors declare that the research was conducted in the absence of any commercial or financial relationships that could be construed as a potential conflict of interest.

## Supplementary Material

Supplementary Figure 1 Flowering time of *Arabidopsis* Col-0 plants grown under SD **(A)** and LD **(B)** conditions.

Supplementary Figure 2 Effects of nitrate availability on the growth of *Arabidopsis* Col-0 plants.

Supplementary Figure 3 Effects of photoperiod on the bolting time of *Arabidopsis* Col-0 plants under a wide range of nitrate supplies.

Supplementary Figure 4 Effects of nitrate availability on the flowering time of *Arabidopsis* Col-0 plants in the soil system.

Supplementary Figure 5 Effects of nitrate availability on the expression of stress-related marker genes under both SD and LD conditions.

Supplementary Figure 6 Effects of ammonium availability on the flowering time of *Arabidopsis* Col-0 plants.

Supplementary Figure 7 Effects of the dysfunction of nitrate assimilation on the flowering response to nitrate availability.

Supplementary Figure 8 Effects of photoperiod on the expression of nitrate uptake- and assimilation-related genes.

Supplementary Figure 9 Disruption of *NRT1.1* and *NRT2.1* functions in *nrt1.1-1/2.1-2* mutants in the soil system delayed flowering.

Supplementary Figure 10 The flowering response of *nrt1.1-1/2.1-2* mutants to nitrate availability in agar system.

Supplementary Figure 11 Flowering time of grafted plants under 1 mM nitrate conditions.

Supplementary Figure 12 Expression of flowering-related genes in both Col-0 plants and *nrt1.1-1/2.1-2* mutants.

Supplementary Table 1 Primers used in quantitative real-time polymerase chain reaction.

Supplementary Table 2 Effects of nitrate availability on the number of rosette leaves of *ft-10* and *co-1* mutants.

Supplementary Table 3 Effects of nitrate availability on flowering of the *cry1* mutants.
