# Supplementary material for: Improved Plant Nitrate Status Involves in Flowering Induction by Extended Photoperiod
Source: Front Plant Sci. 2021 Feb 12;12:629857. doi: 10.3389/fpls.2021.629857 (PMC7907640; doi:10.3389/fpls.2021.629857)
Supplement: Supplementary Figure 1 — Flowering time of Arabidopsis Col-0 plants grown under SD (A) and LD (B) conditions. [file Data_Sheet_1.PDF]

Table 1 Primers used in quantitative real-time polymerase chain reaction.

Gene	Primer	Sequence (5’- 3’)
AT4G05320	*UBQ10* F	ACCCTAACGGGAAAGACGA
	UBQ10 R	GGAGCCTGAGAACAAGATGAA
AT1G12110	*NRT1.1* F	GCACATTGGCATTAGGCTTT
	NRT1.1 R	CTCAATCCCCACCTCAGCTA
AT1G08090	*NRT2.1* F	AACAAGGGCTAACGTGGATG
	NRT2.1 R	CTGCTTCTCCTGCTCATTCC
AT1G65480	*FT* F	CTACAACTGGAACAACCTTTGG
	FT R	TGACAATTGTAGAAAACTGCGG
AT5G15840	*CO* F	GGAGATAGAGTTGTTCCGCTTA
	CO R	CCATGGATGAAATGTATGCGTT
At1g69850	*NRT1.2* F	GTATTTGGCGAATGCGAGTAAT
	NRT1.2 R	TTGAAAAGTGGAGAAGAAAGCG
At1g08100	*NRT2.2* F	CGGAGCACTATTATGTTGGC
	NRT2.2 R	GGTTGCGTTCCCTTTGT
AT5G60770	*NRT2.4* F	GAACAAGGGCTGACATGGAT
	NRT2.4 R	GCTTCTCGGTCTCTGTCCAC
AT1G12940	*NRT2.5* F	GATCTTTTCGGTCCACGTCTAG
	NRT2.5 R	AATCTCACCATGATAAACCCGA
AT1G22770	*GI* F	CTGTTTAAACTGGGAAGCTCAC
	GI R	GGGACAAGGATATAGTACAGCC
AT1G68050	*FKF1* F	GATGATGGAACCATTACACACG
	FKF1 R	TTTGAACACAGGATACGAGACA
AT2G32950	*COP1* F	GTTGCTTGAGTTGGAATAAGCA
	COP1 R	AAACTTTAACCTTGCAGTCGTC
AT4G08920	*CRY1* F	GTTGTTAAATCCACTGGTGCTT
	*CRY1*R	CATAGAGAAAGGACGGCCTAAT
AT5G52310	*RD29A* F	TTCTGTAAGGACGACGTTTACA
	RD29A R	CGTACTCGTTACATCCTCTGTT
AT5G52300	*RD29B* F	GAAACCAAAGATGAGTCGACAC
	RD29B R	TTTTTCGTAAACCGGAGTCAAC
AT5G15960	*KIN1* F	CAAGAATGCCTTCCAAGCCG
	KIN1 R	TGACCCGAATCGCTACTTGTTC
At1g56650	*PAP1* F	GCTCTGATGAAGTCGATCTTC
	PAP1 R	CTACCTCTTGGCTTTCCTCT

Table 2 Effects of nitrate availability on the number of rosette leaves of the *ft-10* and *co-1* mutants.

Rosette leaves	0.2 mM	0.5 mM	1.0 mM	2.0 mM
Col-0	8.1 ± 0.2hi	8.5 ± 0.3h	9.8 ± 0.4g	11.4 ± 0.4f
ft-10	12.6 ± 0.7de	13.2 ± 0.4d	14.4 ± 0.5c	15.6 ± 0.8b
co-1	13.3 ± 0.4d	15.8 ± 0.4b	17.8 ± 0.7ab	18.4 ± 0.5a

Flowering of plants grown with different nitrate concentrations, were determined in an agar system under LD conditions (16-h light/8-h dark). Data represent means ± standard deviations (n = 20). Different letters indicate significant differences between means as determined using a two-way ANOVA followed by a Tukey's multiple comparisons test ($P<0.05$).

Table 3 Effects of nitrate availability on flowering of the *cry1* mutants.

A

Days to bolting	0.2 mM	0.5 mM	1.0 mM	2.0 mM
Col-0	72.5 ± 1.9A	67.4 ± 2.2B	54.4 ± 0.7C	46.0 ± 0.7D
cry1	76.3 ± 2.7a	76.3 ± 2.2a	73.6 ± 3.4a	72.1 ± 2.8a

Days to flowering	0.2 mM	0.5 mM	1.0 mM	2.0 mM
Col-0	80.5 ± 1.8A	76.1 ± 2.0B	61.4 ± 1.0C	51.9 ± 0.8D
cry1	83.6 ± 1.6a	82.9 ± 1.4a	81.4 ± 1.2a	80.3 ± 1.8a

Rosette leaves	0.2 mM	0.5 mM	1.0 mM	2.0 mM
Col-0	13.8 ± 0.6D	15.8 ± 0.8C	17.2 ± 0.5B	20.9 ± 0.5A
cry1	12.2 ± 0.9c	15.2 ± 1.1bc	17.5 ± 0.9ab	18.9 ± 1.0a

B

Days to bolting	0.2 mM	0.5 mM	1.0 mM	2.0 mM
Col-0	24.2 ± 0.9A	20.8 ± 0.7B	18.8 ± 0.8C	16.4 ± 0.3D
cry1	25.8 ± 0.5a	25.6 ± 0.4a	25.4 ± 0.4a	25.1 ± 0.5a

Days to flowering	0.2 mM	0.5 mM	1.0 mM	2.0 mM
Col-0	30.2 ± 1.0A	26.4 ± 0.9B	23.9 ± 1.4C	21.8 ± 0.5D
cry1	33.1 ± 0.5a	31.8 ± 0.4a	31.0 ± 0.3a	31.4 ± 1.1a

Rosette leaves	0.2 mM	0.5 mM	1.0 mM	2.0 mM
Col-0	8.0 ± 0.1D	8.6 ± 0.2C	9.9 ± 0.4B	11.6 ± 0.2A
cry1	7.3 ± 0.2c	7.9 ± 0.3bc	8.4 ± 0.3ab	9.2 ± 0.6a

Flowering of plants grown with different nitrate concentrations, were determined in an agar system under SD (A) and LD (B) conditions. SD: 8-h light/ 16-h dark; LD: 16-h light/ 8-h dark. Data represent means ± standard deviations (n = 20). Different letters indicate significant differences between means as determined using a one-way ANOVA followed by a Tukey's multiple comparisons test (P<0.05).

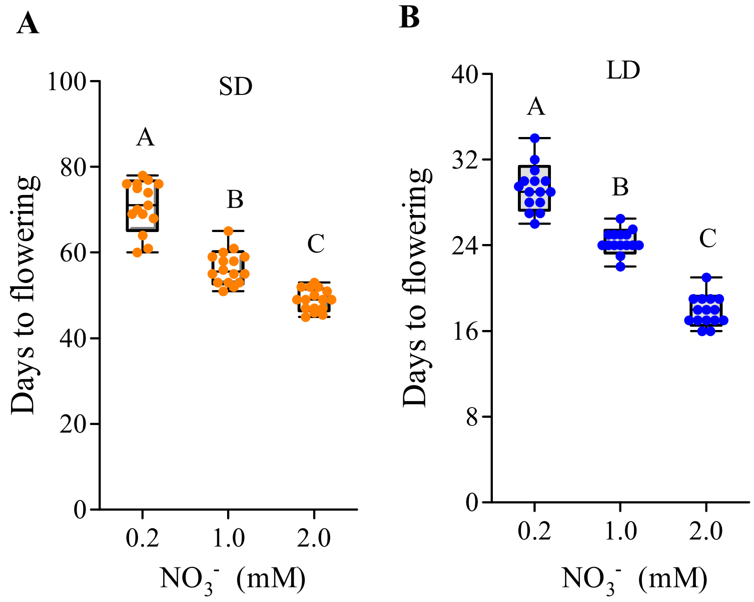

Fig. S1 Flowering time of *Arabidopsis* Col-0 plants grown under SD (A) and LD (B) conditions. Seedlings were planted in media containing 0.2, 1, or 2 mM nitrate. The potassium concentration in all growth media was adjusted to 2 mM using K_2SO_4. SD: 8-h light/ 16-h dark; LD: 16-h light/ 8-h dark. At least 20 plants grown in each of the LD and SD conditions were used to determine the flowering time. Error bars represent ± standard deviations (n = 15). Different letters indicate significant differences between means, as determined using a one-way ANOVA followed by a Tukey's multiple comparisons test (P<0.05).

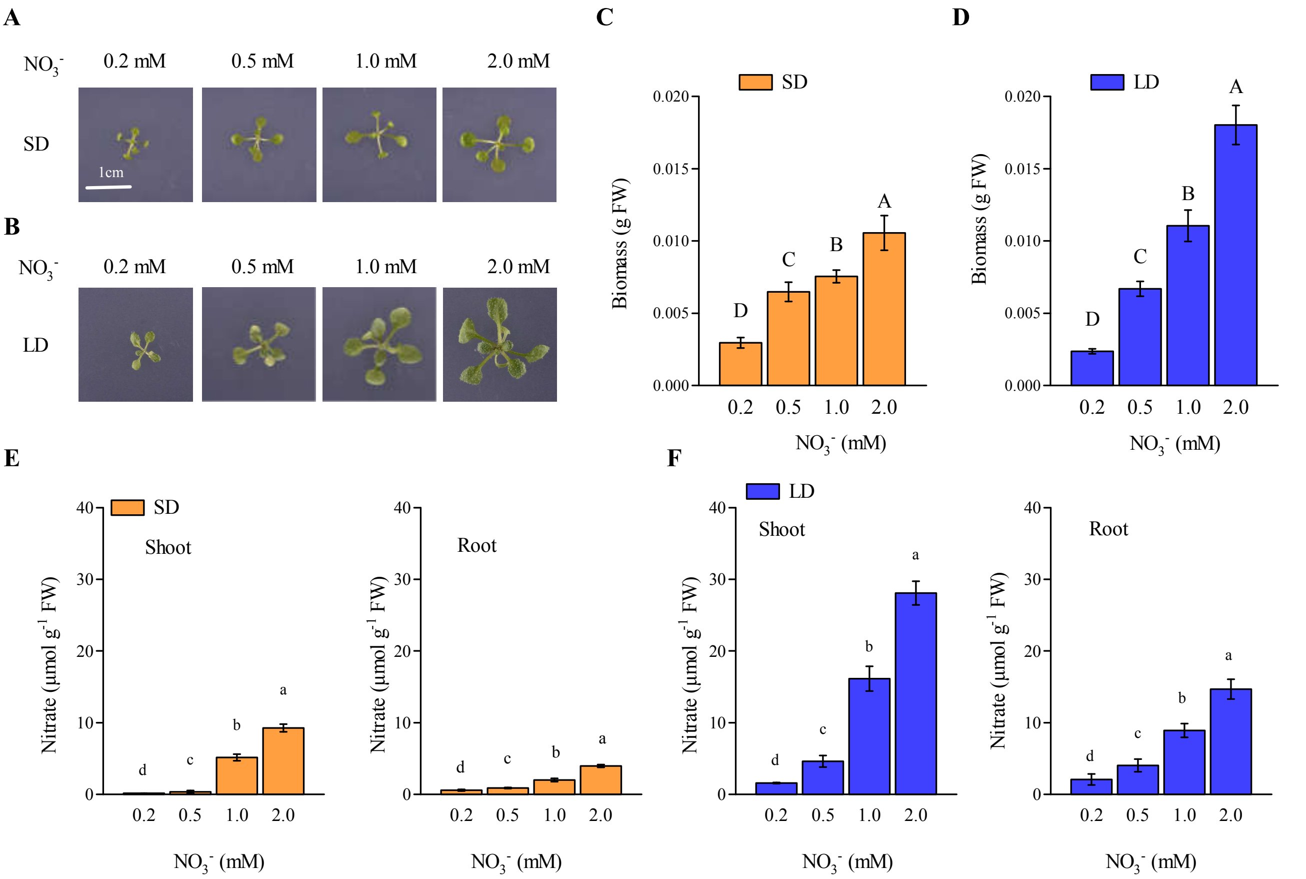

Fig. S2 Effects of nitrate availability on the growth and internal nitrate level of *Arabidopsis* Col-0 plants. The phenotypic photographs (A) and biomass (C) of Col-0 plants grown for 16 days under SD conditions. The phenotypic photographs (B) and biomass (D) of Col-0 plants grown for 16 days under LD conditions. The internal nitrate level of Col-0 plants grown under both SD (E) and LD conditions (F). The plants were grown in agar system containing different nitrate concentrations as the sole N source. SD: 8-h light/ 16-h dark; LD: 16-h light/ 8-h dark. Error bars represent ± standard deviations (n = 12). Different letters indicate significant differences between means as determined using a one-way ANOVA followed by a Tukey's multiple comparisons test (P<0.05).

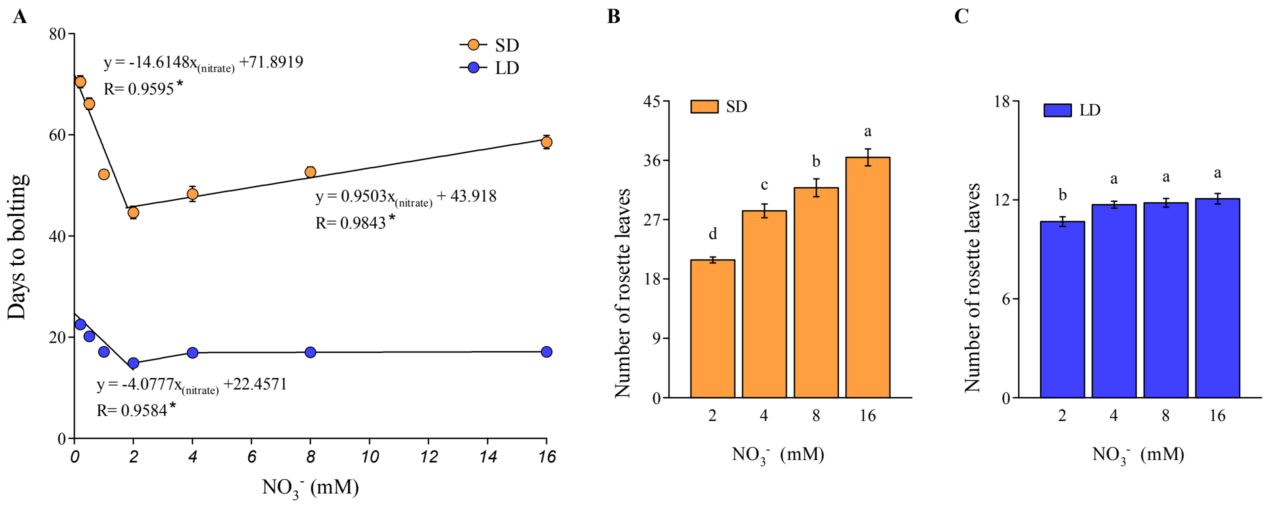

Fig. S3 Effects of photoperiod on the bolting time of *Arabidopsis* Col-0 plants under a wide range of nitrate supplies. (A) Days to bolting in Col-0 plants under both SD and LD conditions. Number of rosette leaves of Col-0 plants treated with high nitrate concentrations under SD (B) and LD (C) conditions. Seedlings were grown on agar medium containing a series of KNO_3 concentrations, as indicated. SD, 8-h light/ 16-h dark; LD: 16-h light/ 8-h dark. Error bars represent ± standard deviations (n = 20). Different letters indicate significant differences between means as determined using a one-way ANOVA followed by a Tukey's multiple comparisons test (P<0.05).

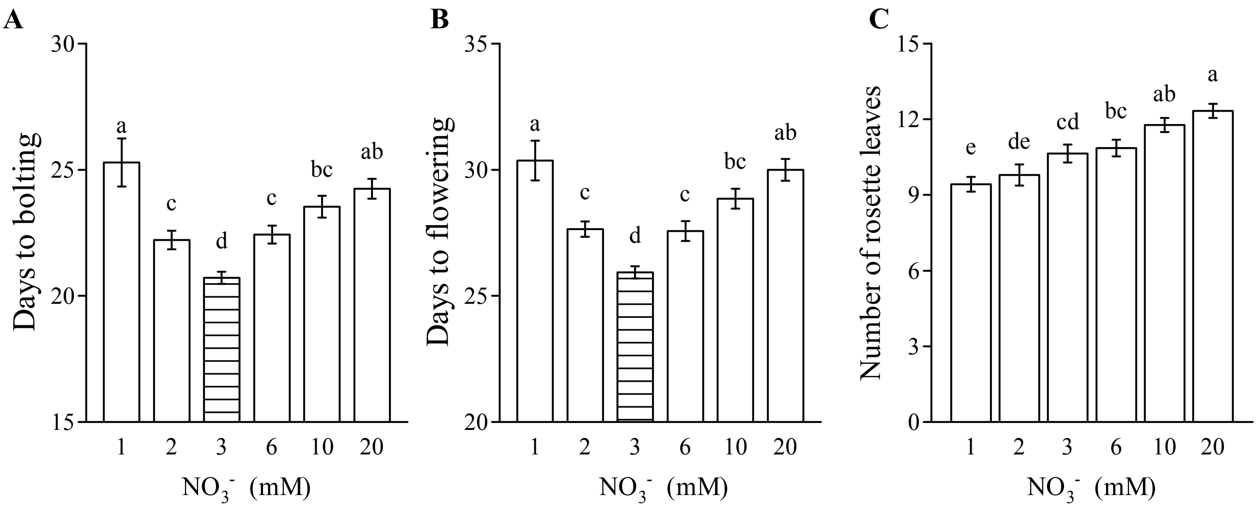

Fig. S4 Effects of nitrate availability on the flowering time of *Arabidopsis* Col-0 plants in the soil system. Days to bolting (A), days to flowering (B), and number of rosette leaves (C)were determined in plants were treated with different nitrate concentrations under LD conditions. LD, 16-h light/ 8-h dark. Error bars represent ± standard deviations (n = 20). Different letters indicate significant differences between means as determined using a one-way ANOVA followed by a Tukey's multiple comparisons test (P<0.05).

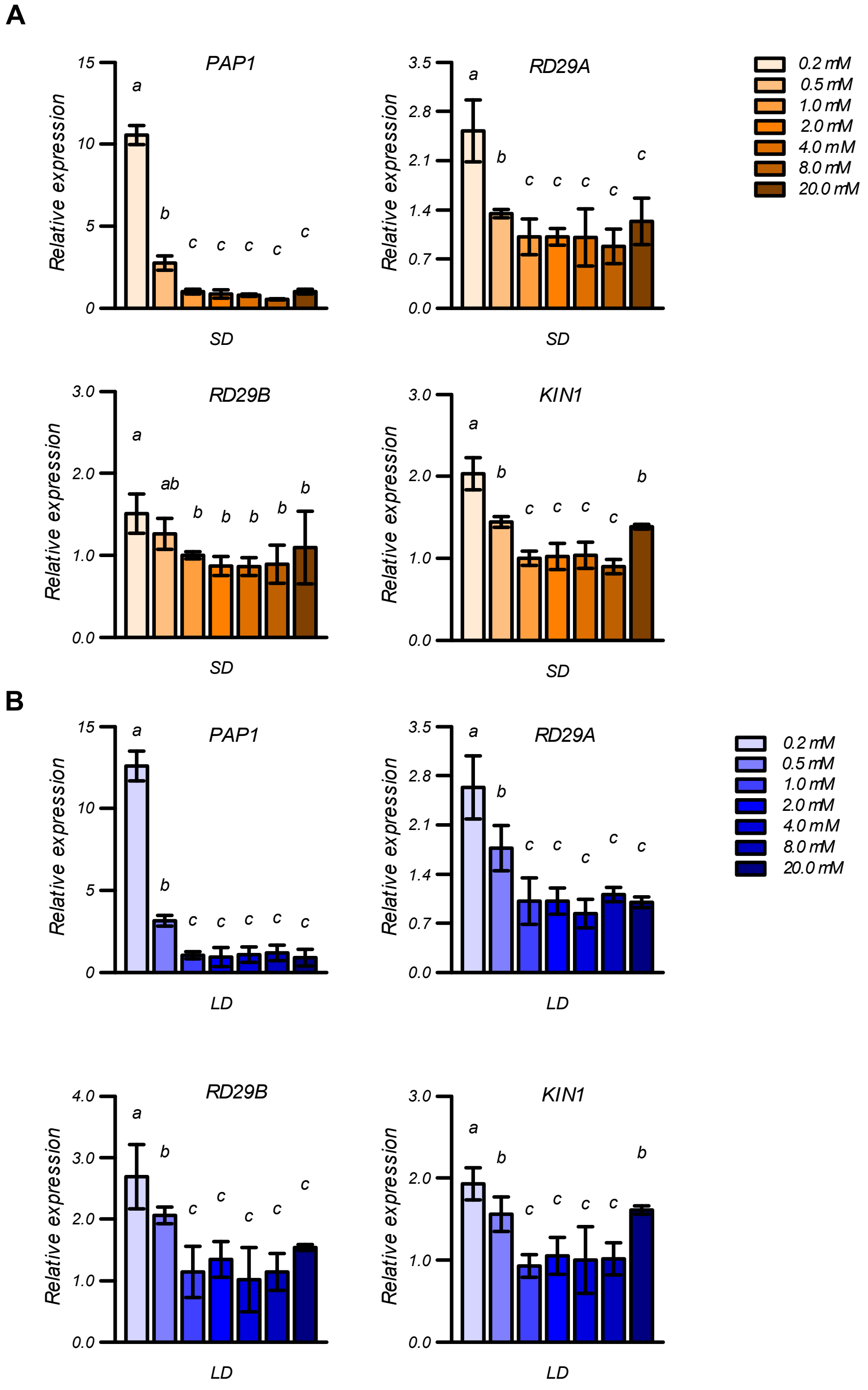

Fig. S5 Effects of nitrate availability on the expression of stress-related marker genes under SD and LD conditions. The Col-0 seedlings (at the stage when six leaves were visible) grown in agar medium containing a series of KNO_3 concentrations were harvested for RNA extraction at ZT 16 h. The *UBIQUITIN 10* gene was used as an internal reference. SD: 8-h light/ 16-h dark; LD: 16-h light/ 8-h dark. Error bars represent ± standard deviations (n = 4). Different letters indicate significant differences between means as determined using a one-way ANOVA followed by a Tukey's multiple comparisons test (*P*<0.05).

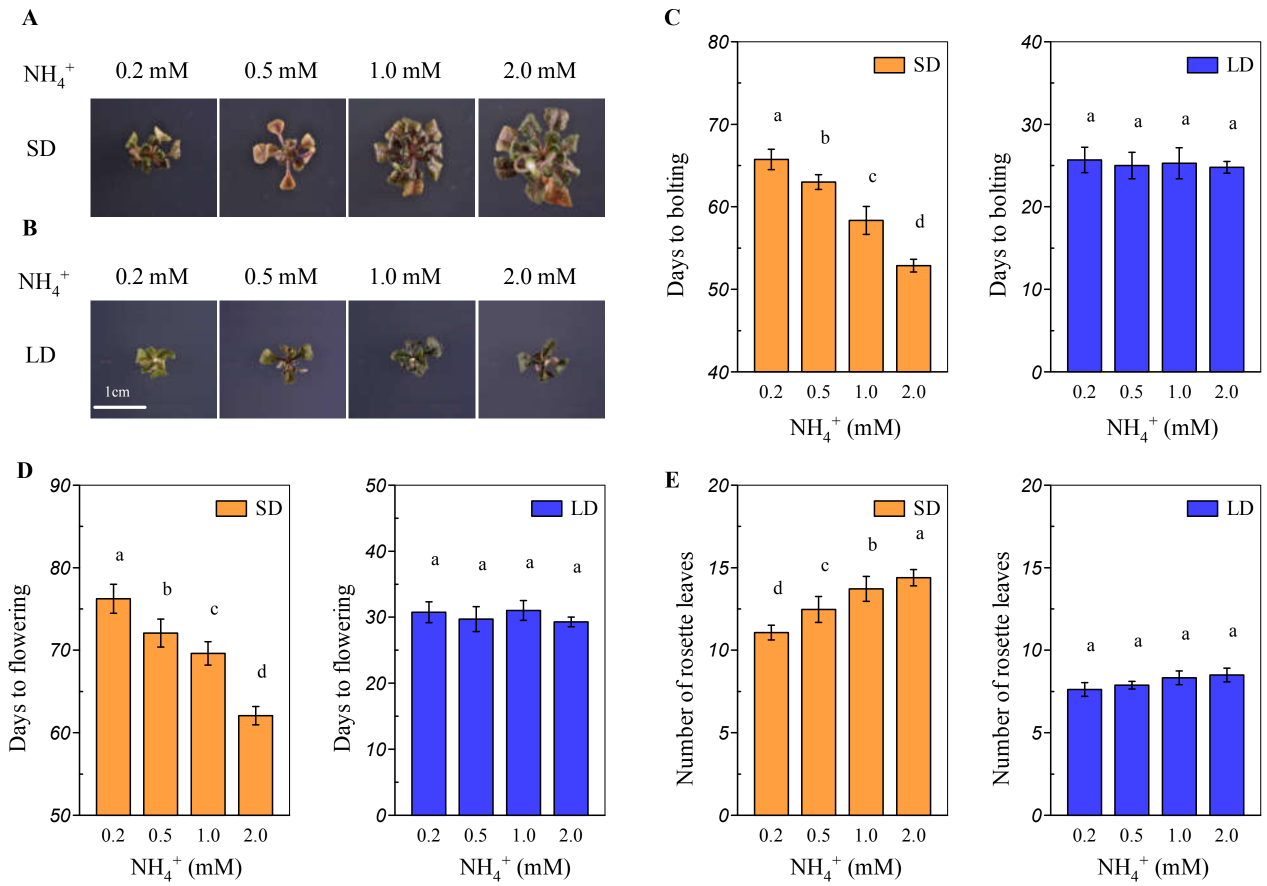

Fig. S6 Effects of ammonium availability on the flowering time of *Arabidopsis* Col-0 plants. The phenotypic photographs of the flowering of the plants treated with different nitrate concentrations under both SD (A) and LD (B) conditions. Days to bolting(C), days to flowering (D), and number of rosette leaves (E) in Col-0 plants treated with different ammonium concentrations under both SD and LD conditions. The surface-sterilized seeds were sown on agar medium containing a range of concentrations of $(NH_4)_2SO_4$ as indicated. To minimize the effect of ammonium toxicity on the growth and flowering of plants, 2.5mM MES was supplied and the pH of the agar base was adjusted to 6.5 at the beginning of sowing. SD, 8-h light/ 16-h dark; LD: 16-h light/ 8-h dark. Error bars represent ± standard deviations (n = 20). Different letters indicate significant differences between means as determined using a one-way ANOVA followed by a Tukey's multiple comparisons test (P<0.05).

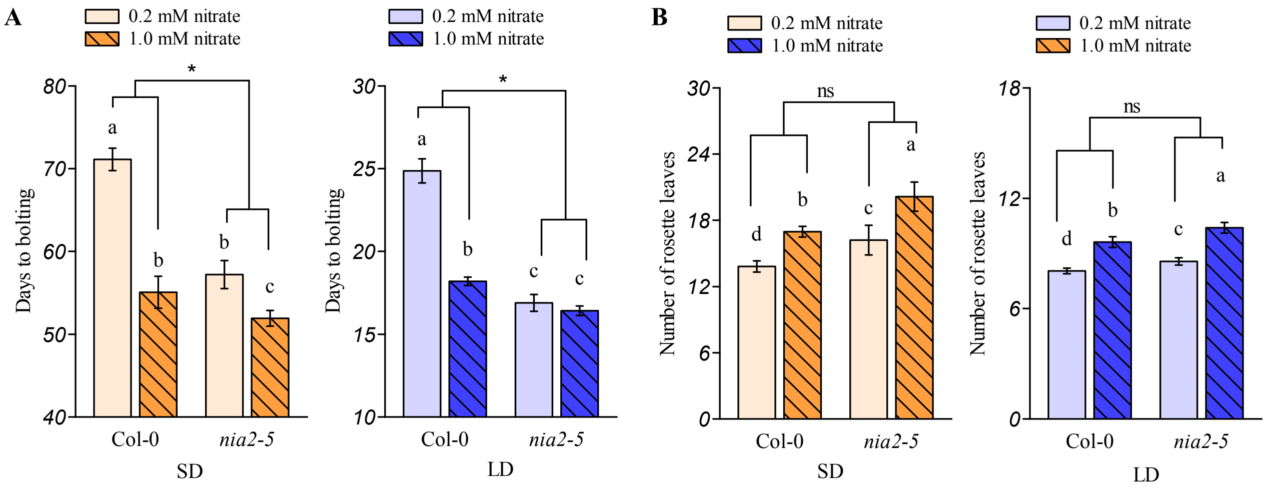

Fig. S7 Effects of the dysfunction of nitrate assimilation on the flowering response to nitrate availability. Seedlings were grown on agar media containing nitrate as the sole N sources under both SD (A) and LD (B) conditions. The potassium concentration in all growth media was adjusted to 1 mM using K_2SO_4. SD: 8-h light/ 16-h dark; LD: 16-h light/ 8-h dark. Error bars represent ± standard deviations (n = 15). Different letters indicate significant differences between means as determined using a two-way ANOVA followed by a Tukey's multiple comparisons test (*P*<0.05).

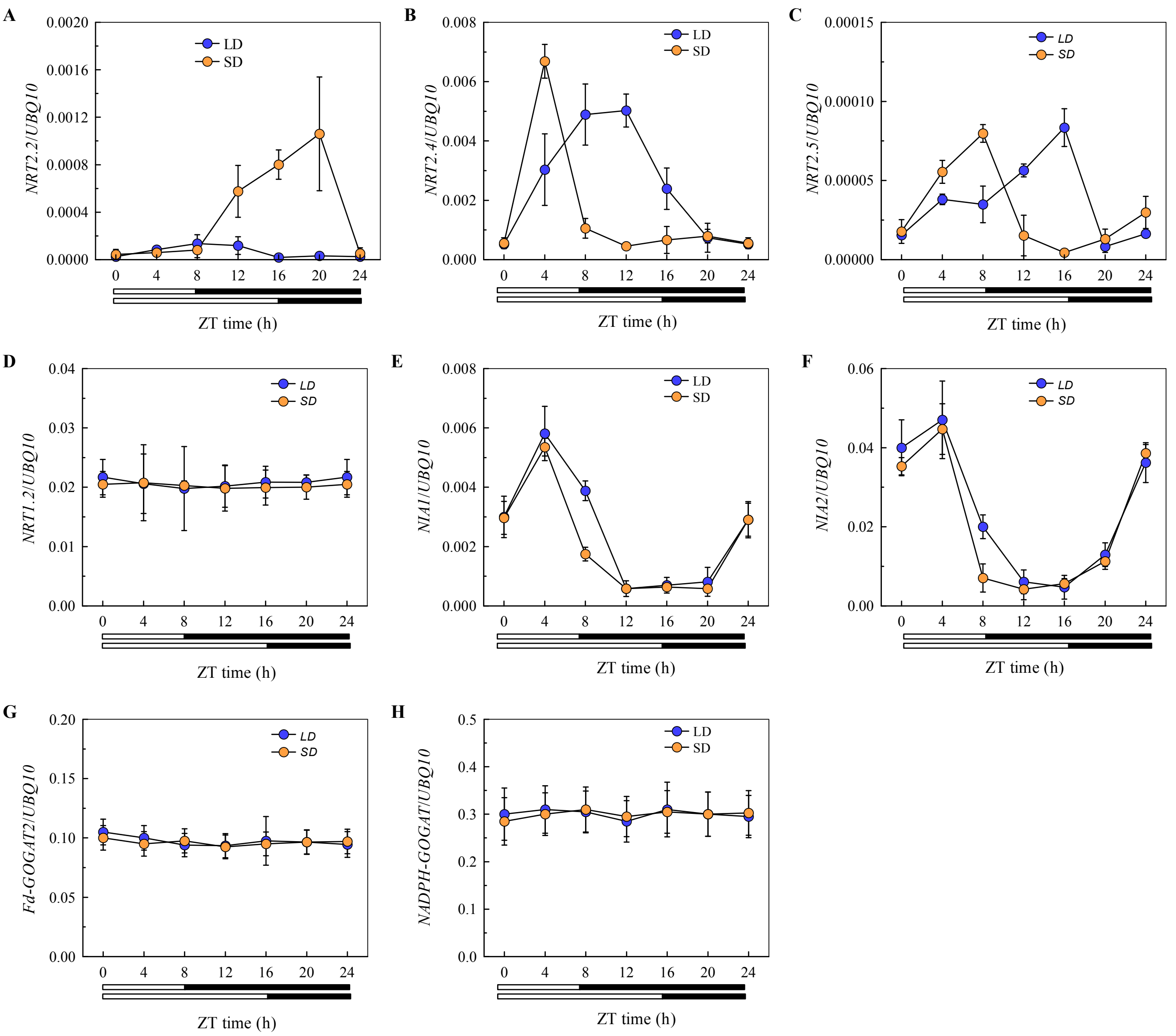

Fig. S8 Effects of photoperiod on the expression of nitrate uptake- and assimilation-related genes. The *mRNA* level of genes involved in nitrate uptake (*NRT2.2*, *NRT2.4*, *NRT2.5*, *NRT1.2*) and nitrate assimilation (*NIA1*, *NIA2*, *Fd-GOGAT2*, *NADPH-GOGAT*) were determined in the roots of Col-0 plants under both LD and SD photoperiods. Seven-day-old seedlings grown in agar medium with 1 mM KNO_3 were harvested. Bars below the graph indicated the duration of day (white) and night (black). Expression levels were normalized to the expression of *UBIQUITIN 10*. Error bars represent ± standard deviations (n = 4). SD, 8-h light/ 16-h dark; LD: 16-h light/ 8-h dark.

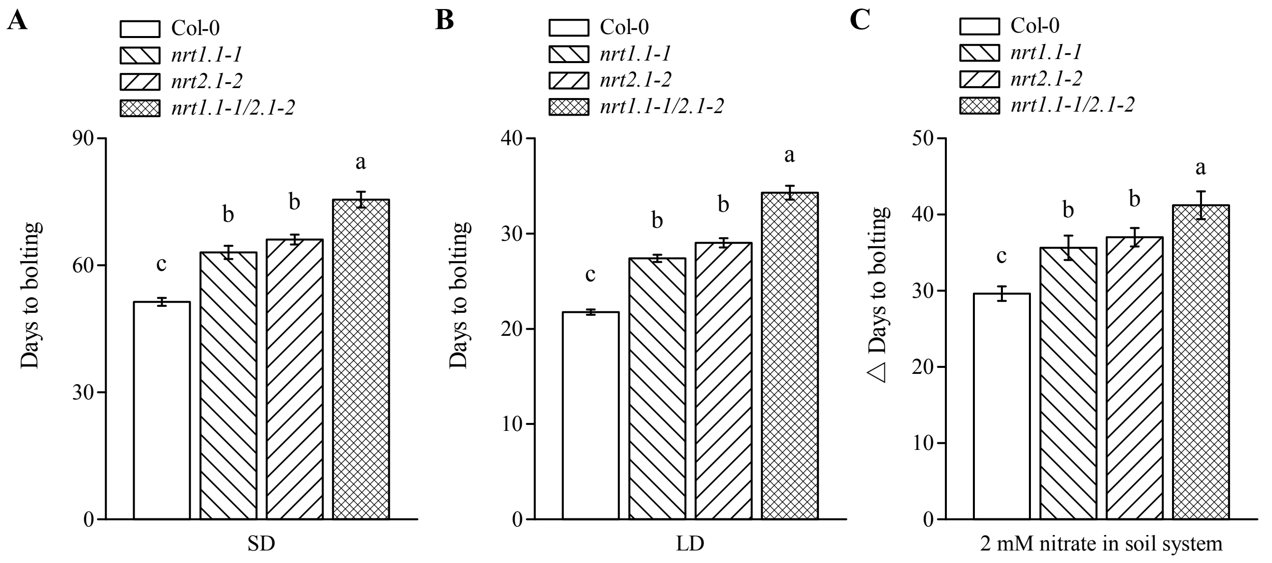

Fig. S9 Disruption of *NRT1.1* and *NRT2.1* functions in *nrt1.1-1/2.1-2* mutants in the soil system delayed flowering. Days to bolting of the Col-0, *nrt1.1-1*, *nrt2.1-2*, and *nrt1.1-1/2.1-2* plants grown under SD (A) and LD (B) conditions. (C) The time of LD-induced flowering (△ days to bolting) in these lines. The plants were grown in a soil system and watered with medium twice a week with approximately 20 mL of medium per pot, per watering event. The composition of the medium for the soil system was as follows: KNO_3 (2 mM), NaH_2PO_4 (1000 μM), $MgSO_4$ (500 μM), $CaCl_2$ (1000 μM), H_3BO_3 (10 μM), $MnSO_4$ (0.5 μM), $ZnSO_4$ (0.5 μM), $CuSO_4$ (0.1 μM), $(NH_4)_6Mo_7O_{24}$ (0.1 μM), and Fe-EDTA (25 μM). △ Days to bolting was calculated by subtracting the bolting time of plants grown under LD conditions from that of plants grown under SD conditions, with the same nitrogen treatment. SD: 8-h light/ 16-h dark; LD: 16-h light/ 8-h dark. At least 20 plants grown in each of the LD and SD conditions were used to determine the flowering time. Error bars represent ± standard deviations (n = 12). Different letters indicate significant differences between means as determined using a one-way ANOVA followed by Tukey's multiple comparisons test ($P<0.05$).

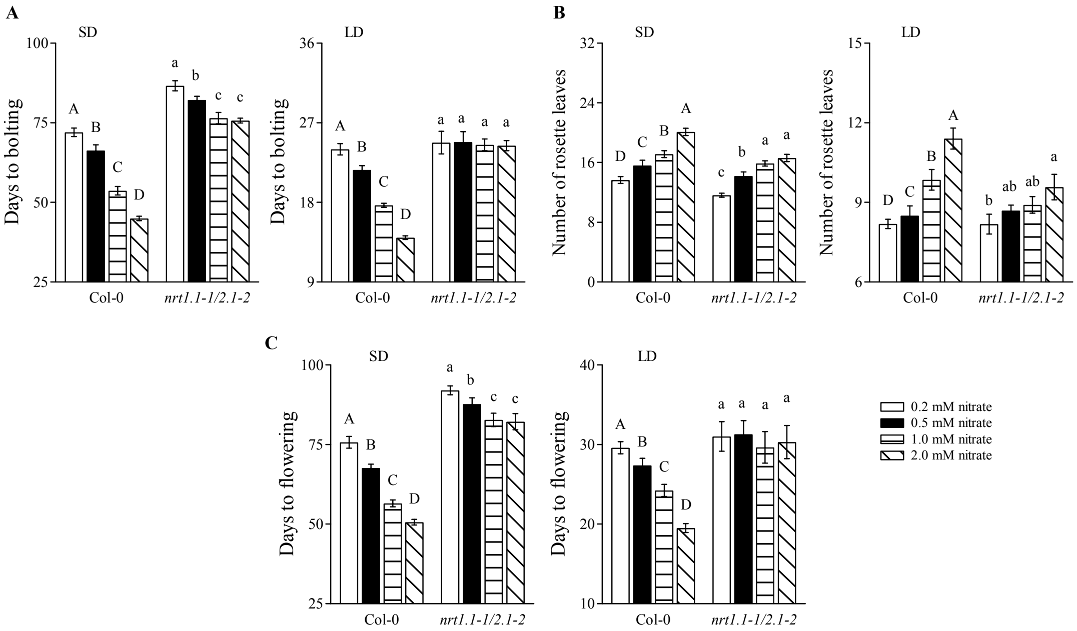

Fig. S10 Flowering responses of *nrt1.1-1/2.1-2* mutants to nitrate availability in agar system. Days to bolting (A), number of rosette leaves (B) and days to flowering (C) in plants treated with different nitrate concentrations under both SD and LD conditions. Seedlings were grown on agar medium containing a series of KNO_3 concentrations as indicated. The potassium concentration in all growth media were adjusted to 2 mM using K_2SO_4. SD: 8-h light/ 16-h dark; LD: 16-h light/ 8-h dark. At least 20 plants grown in each of the LD and SD conditions were used to determine the flowering time. Error bars represent ± standard deviations (n = 12). Different letters indicate significant differences between means as determined using a one-way ANOVA followed by Tukey's multiple comparisons test (P<0.05).

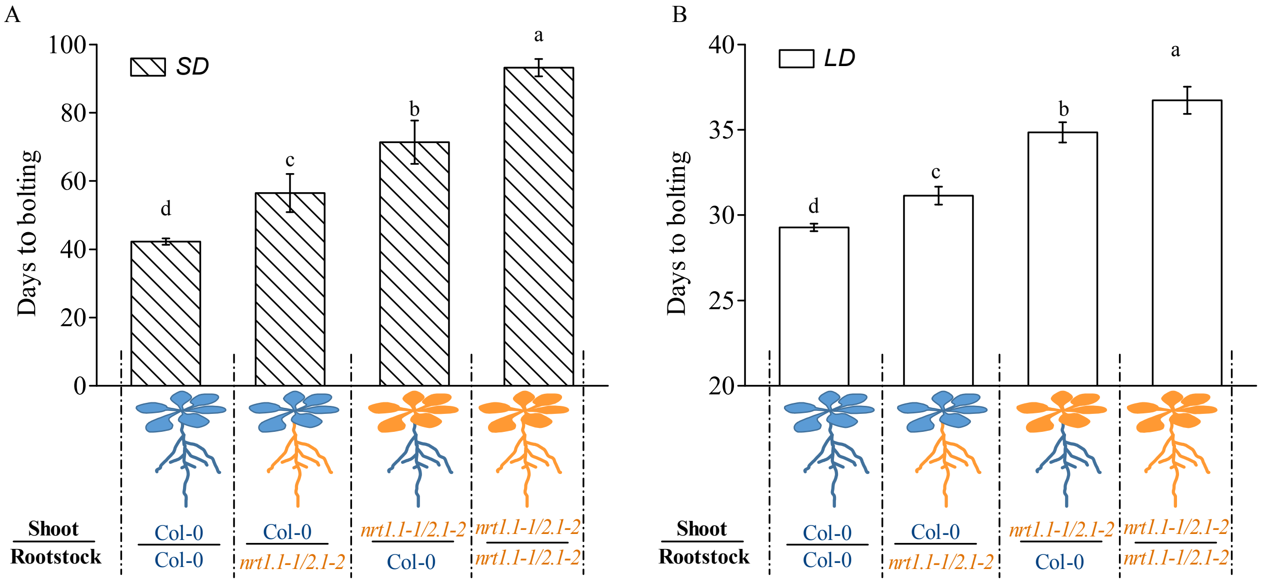

Fig.S11 Flowering time of grafted plants under 1 mM nitrate conditions. Grafted plants grown in agar system were used to determine the bolting time under SD (A) and LD (B) conditions. SD: 8-h light/ 16-h dark; LD: 16-h light/ 8-h dark. Error bars represent ± standard deviations (n = 6). Different letters indicate significant differences between means as determined using a one-way ANOVA followed by a Tukey's multiple comparisons test (P<0.05).

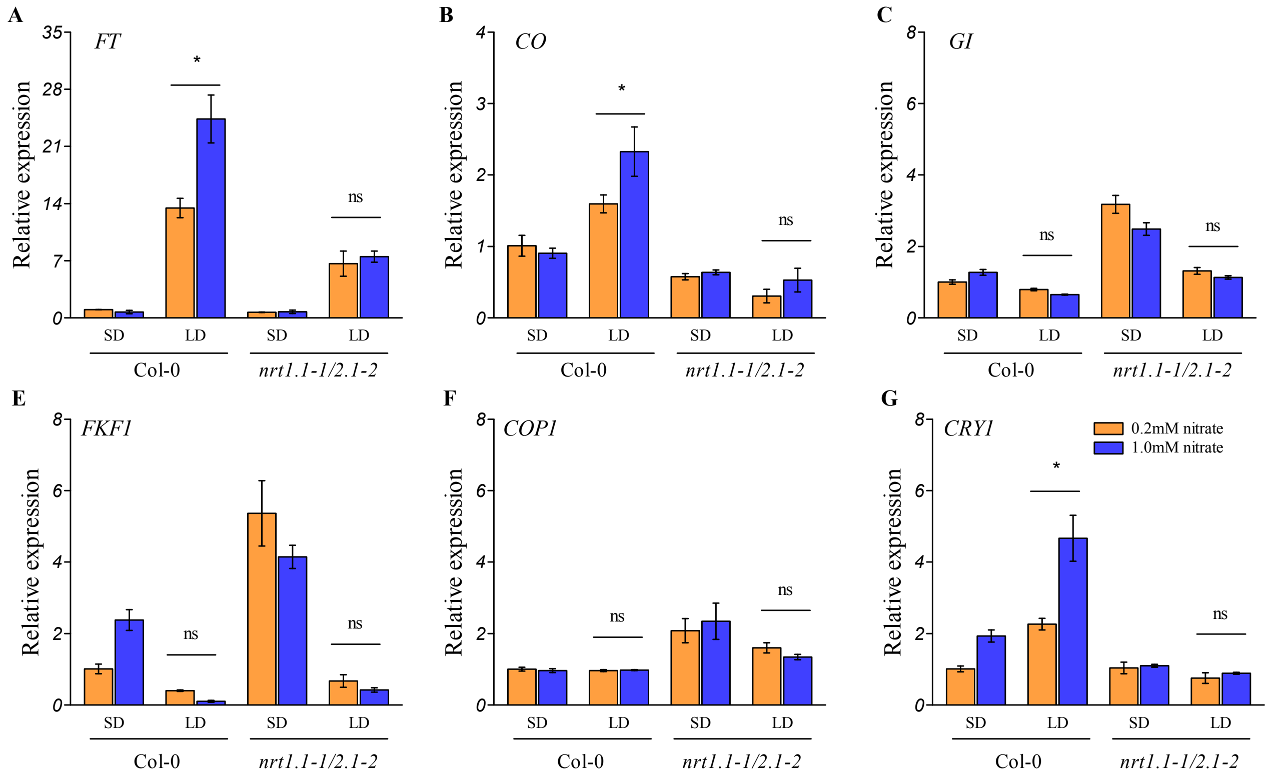

Fig. S12 Expression of flowering-related genes in both Col-0 plants and *nrt1.1-1/2.1-2* mutants. The mRNA level of *FT* (A), *CO* (B), *GI*(C), *FKF1* (D), *COP1* (E) and *CRY1* (G) in shoots of the Col-0 and *nrt1.1-1/2.1-2* plants, was determined under LD conditions. The plants (at the stage when four leaves were visible) grown in agar medium containing either 0.2 or 1.0 mM nitrate were harvested at ZT 16 h. The potassium concentration in all growth media was adjusted to 1 mM using K_2SO_4. Expression levels were normalized to the expression of *UBIQUITIN 10*. SD: 8-h light/ 16-h dark; LD: 16-h light/ 8-h dark. Error bars represent ± standard deviations (n = 4). Significant differences were determined using a two-tailed Student's t-tests: *, P<0.05; ns, non-significant.
